# Dissection of insular cortex layer 5 reveals two sublayers with opposing modulatory roles in appetitive drinking behavior

**DOI:** 10.1016/j.isci.2023.106985

**Published:** 2023-05-26

**Authors:** Makoto Takemoto, Shigeki Kato, Kazuto Kobayashi, Wen-Jie Song

**Affiliations:** 1 Department of Sensory and Cognitive Physiology, Faculty of Life Sciences, Kumamoto University, Kumamoto 860-8556, Japan; 2 Department of Molecular Genetics, Institute of Biomedical Sciences, Fukushima Medical University School of Medicine, Fukushima 960-1295, Japan; 3 Center for Metabolic Regulation of Healthy Aging, Faculty of Life Sciences, Kumamoto University, Kumamoto 860-8556, Japan

**Keywords:** Neuroscience, Behavioral neuroscience

## Abstract

The insular cortex (insula) is known to play a modulatory role in feeding and drinking. Previous studies have revealed anterior-posterior differences of subcortical projections and roles for the insula, yet the anatomical and functional heterogeneity among the cortical layers remains poorly understood. Here, we show that layer 5 of the mouse dysgranular insula has two distinct neuronal subpopulations along the entire anterior-posterior axis: The L5a population, expressing NECAB1, projects bilaterally to the lateral and capsular divisions of the central amygdala, and the L5b population, expressing CTIP2, projects ipsilaterally to the parasubthalamic nucleus and the medial division of the central amygdala. Optogenetically activating L5a and L5b neuronal populations in thirsty male mice led to suppressed and facilitated water spout licking, respectively, without avoidance against or preference for the spout paired with the opto-stimulation. Our results suggest sublayer-specific bidirectional modulatory roles of insula layer 5 in the motivational aspect of appetitive behavior.

## Highlights

• L5 sublayers of the dysgranular insula have distinct subcortical projections
• The organizations of L5a/L5b projections to the extended amygdala are complementary
• Optogenetic activations of L5 sublayers have opposite effects on appetitive drinking
• The opto-activations have no effect on the induction of emotional valence

## Graphical abstract

**Figure:**
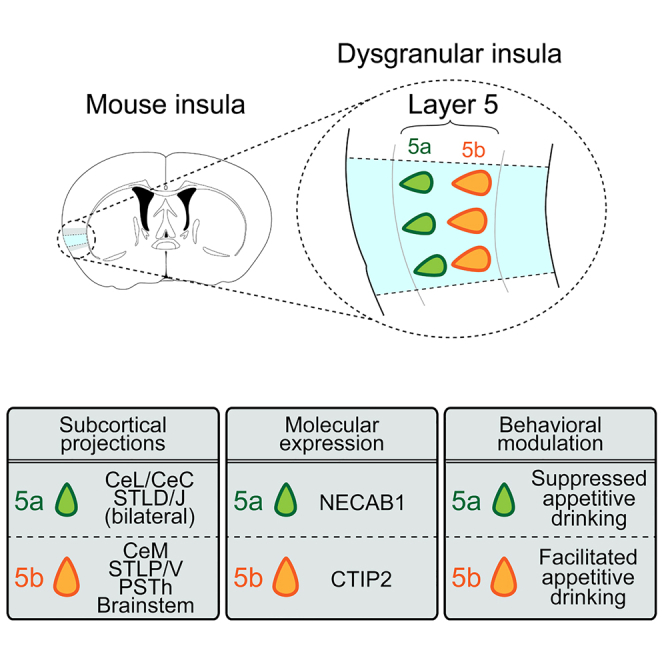

## Introduction

The insular cortex (insula) mediates top-down modulation of feeding and drinking behaviors in response to taste quality,^1^^,^^2^ homeostatic/visceral states,^3^^,^^4^ innate threat,^3^ and learned stimuli,^5^^,^^6^^,^^7^^,^^8^^,^^9^^,^^10^^,^^11^^,^^12^ as well as the modulation of emotional^13^ and social^3^^,^^14^^,^^15^ behaviors. In particular, the modulation of feeding or drinking is differentially regulated by distinct subregions of the insula along the anterior-posterior (A-P) axis,^1^ and region-specific projections to the amygdala complex are suggested to underlie the behavioral control. The projection of the anterior insula to the basolateral amygdala (BLA) and the projection of the posterior insula to the central amygdala (CeA) serve to enhance and suppress drinking, respectively, by inducing opposite emotional valences.^2^ The suppressive effect of the posterior insula-CeA circuit on drinking and feeding behaviors has been consistently observed in several studies.^3^^,^^8^^,^^16^ The region-specific hard-wired connections of the insula have been proposed to underlie the taste quality-dependent control of feeding/drinking as a study has demonstrated that sweet and bitter (i.e., appetitive and aversive) tastes are primarily represented in the anterior and posterior part, respectively, of the gustatory insula,^17^ although the “topographical taste coding” is still controversial as recent studies have demonstrated that taste representations spatially overlap within the insula at a neuronal level.^18^^,^^19^

On the other hand, feeding/drinking behavior is not merely determined by taste quality but is also adaptively regulated by internal and external circumstances.^20^^,^^21^ Therefore, additional neural substrates may also participate in the modulation of feeding/drinking. Indeed, Stern et al.^10^ have demonstrated that the CeA projection from nitric oxide synthase-1-expressing neuronal subpopulation in the middle insula mediates an enhanced feeding in response to a learned context. This observation contrasts the feeding-suppressive effect of the insula-CeA circuit reported in the studies mentioned above, suggesting heterogeneity in neuronal populations of the middle insula that project to the CeA.

Anatomical and functional heterogeneity of subcortical projection neurons of the insula has been investigated intensely in terms of the differences among the subregions.^1^^,^^2^^,^^3^^,^^15^^,^^22^^,^^23^^,^^24^^,^^25^^,^^26^^,^^27^ However, much less attention has been given to the layer specificity that includes neuronal diversity within a layer and among layers, regardless of the unique laminar cytoarchitecture of the insula.^23^^,^^28^ Only a few anatomical studies have been carried out so far, reporting the layer 5 (L5) origin of insula projections to subcortical structures, such as the parabrachial nucleus (PBN) of the pons^29^ and the nucleus of the solitary tract (NTS).^11^^,^^24^^,^^30^^,^^31^

Here, we demonstrate that L5 of the dysgranular insula (DgI) has two distinct neuronal subpopulations along the whole A-P axis in male mice. One, primarily located in the upper L5 (L5a), projects bilaterally to the lateral and capsular divisions of the CeA (CeL/CeC) and suppresses appetitive drinking on its optogenetic activation. The other, predominantly located in the deeper L5 (L5b), projects ipsilaterally to the parasubthalamic nucleus (PSTh) of the hypothalamus and the medial division of the CeA (CeM) and facilitates appetitive drinking on its optogenetic activation. These findings suggest opposite modulatory roles of sublayers of insula L5 in appetitive behavior.

## Results

### Hemispheric difference in insula projections to the extended amygdala and parasubthalamic nucleus

**Figure 1:**
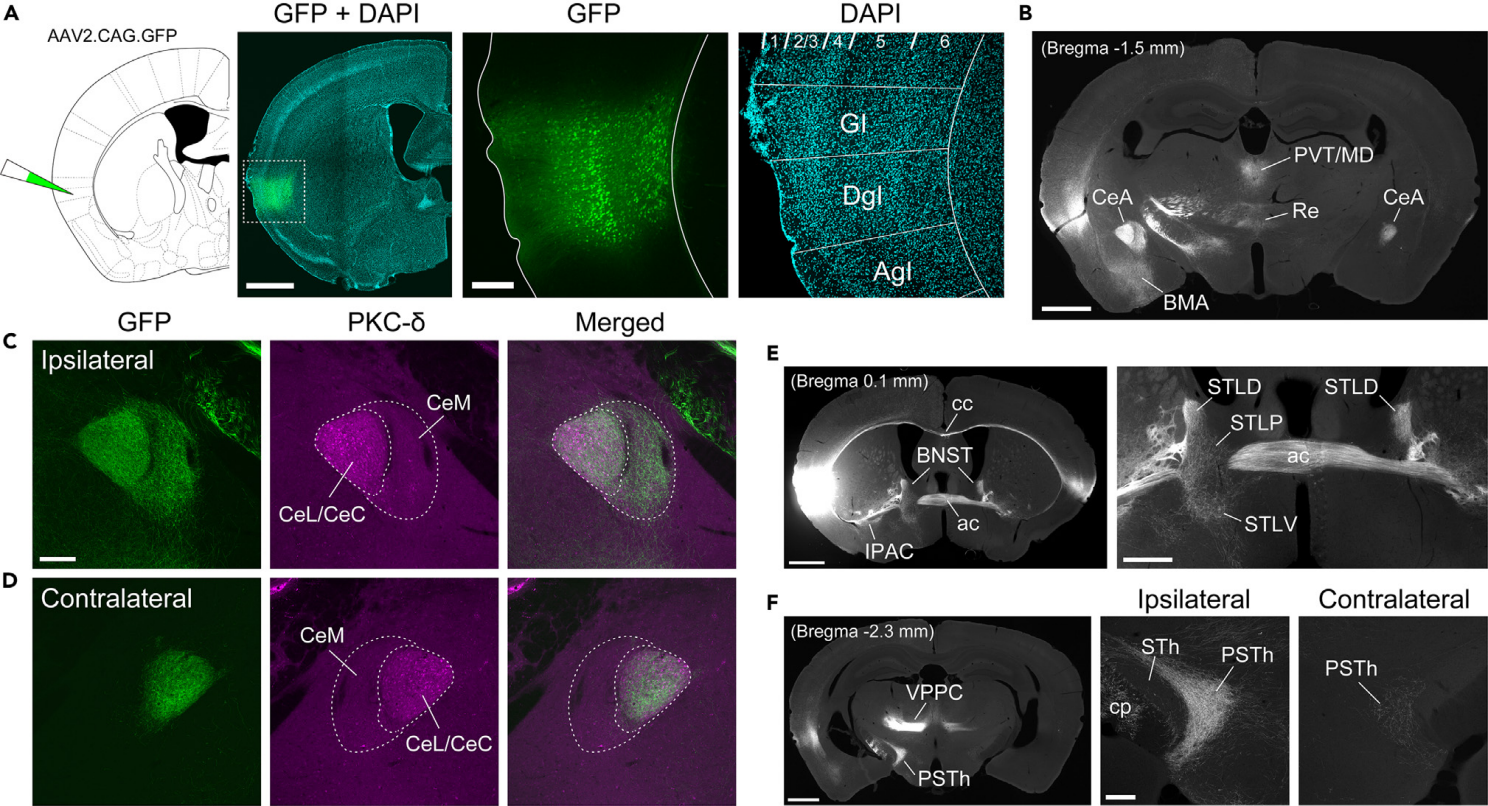
Hemispheric difference in subcortical projections of the insula (A) A typical example of the insula injected with the AAV2.CAG.GFP vector. GFP is expressed primarily in the middle to deep layers of the dysgranular (DgI) and granular (GI) regions of the insula. (B) A gray-scale image of the distribution of GFP-expressing axon arbors in the amygdala and thalamus. (C) GFP-labeled axon arbors in the ipsilateral CeA. The arborization (green) is located in the PKC-δ positive CeL/CeC (magenta) and the adjacent CeM. (D) GFP-labeled axon arbors in the contralateral CeA. The arborization is localized in the PKC-δ positive CeL/CeC. (E) A gray-scale image of GFP-expressing axons in the BNST and ventral striatum (left) and a magnified view of the BNST (right). (F) A gray-scale image of GFP-expressing axon arbors in the PSTh and thalamus (left) and magnified views of the ipsilateral and contralateral PSTh (right). Abbreviations: ac, anterior commissure; AgI, agranular insula; BMA, basomedial amygdala; cc, corpus callosum; cp, cerebral peduncle; IPAC, interstitial nucleus of the posterior limb of the anterior commissure; MD, mediodorsal thalamic nucleus; PVT, paraventricular thalamic nucleus; Re, nucleus reuniens; STh, subthalamic nucleus; VPPC, parvicellular part of the ventral posterior thalamic nucleus. Scale bars: 1 mm (A-left, B and E-left, F-left), 0.2 mm (A-right, C and F-right), 0.5 mm (E-right). See also Figure S1.

To investigate the heterogeneity of the mouse insula subcortical projections, we first labeled neurons in the middle-posterior insula with GFP using the AAV2.CAG.GFP vector. The injection site (0.05 ± 0.3 mm posterior to bregma) with GFP-expressing neuronal somata covered the ventral part of the granular insula (GI), which contains a clear granular layer (i.e., layer 4), and the DgI, with a vague layer 4, located dorsal to the agranular insula (AgI) that lacks a six-layered structure^23^^,^^28^ (Figure 1A). GFP-labeled axon arbors were found in many subcortical structures that include the paraventricular and mediodorsal thalamic nuclei, nucleus reuniens, CeA, basomedial amygdala (BMA) (Figure 1B), bed nucleus of the stria terminalis (BNST), interstitial nucleus of the posterior limb of the anterior commissure (Figure 1E), parvicellular part of the ventral posterior thalamic nucleus, and PSTh (Figure 1F), as well as the nucleus accumbens, periaqueductal gray, PBN, and NTS (Figures S1B–S1E), in line with the results of previous anatomical studies.^2^^,^^10^^,^^22^^,^^23^^,^^27^^,^^32^ There were virtually no retrogradely labeled cell bodies in these structures.

Focusing on the dense labeling of GFP-positive axons projecting to the CeA of each hemisphere, we found the projection patterns to be different between the hemispheres. The identification of the CeL/CeC with a molecular marker, the protein kinase C delta (PKC-δ),^33^ revealed that the ipsilateral projection was distributed in both the CeL/CeC and adjacent CeM (Figure 1C), whereas the contralateral projection was confined to the CeL/CeC (Figure 1D). Such asymmetric projections were also found in the lateral division of the BNST (STL), in which the ipsilateral projections were seen in the dorsal (STLD, also referred to as the oval nucleus of the BNST), posterior (STLP), and ventral (STLV) part of the STL, whereas the contralateral projection was found only in the STLD (Figure 1E). In the hypothalamus, dense axon arbors expressing GFP were packed in the ipsilateral PSTh, but few labeled fibers were found on the contralateral side (Figure 1F). The same patterns of hemispheric projections were seen for both left and right insulae (Figures S1F and S1G). These findings indicate that unilateral and bilateral subcortical projections of the insula are organized in a target-dependent fashion.

### Sublaminar and regional distribution of anatomically distinct neuronal populations in DgI L5

**Figure 2:**
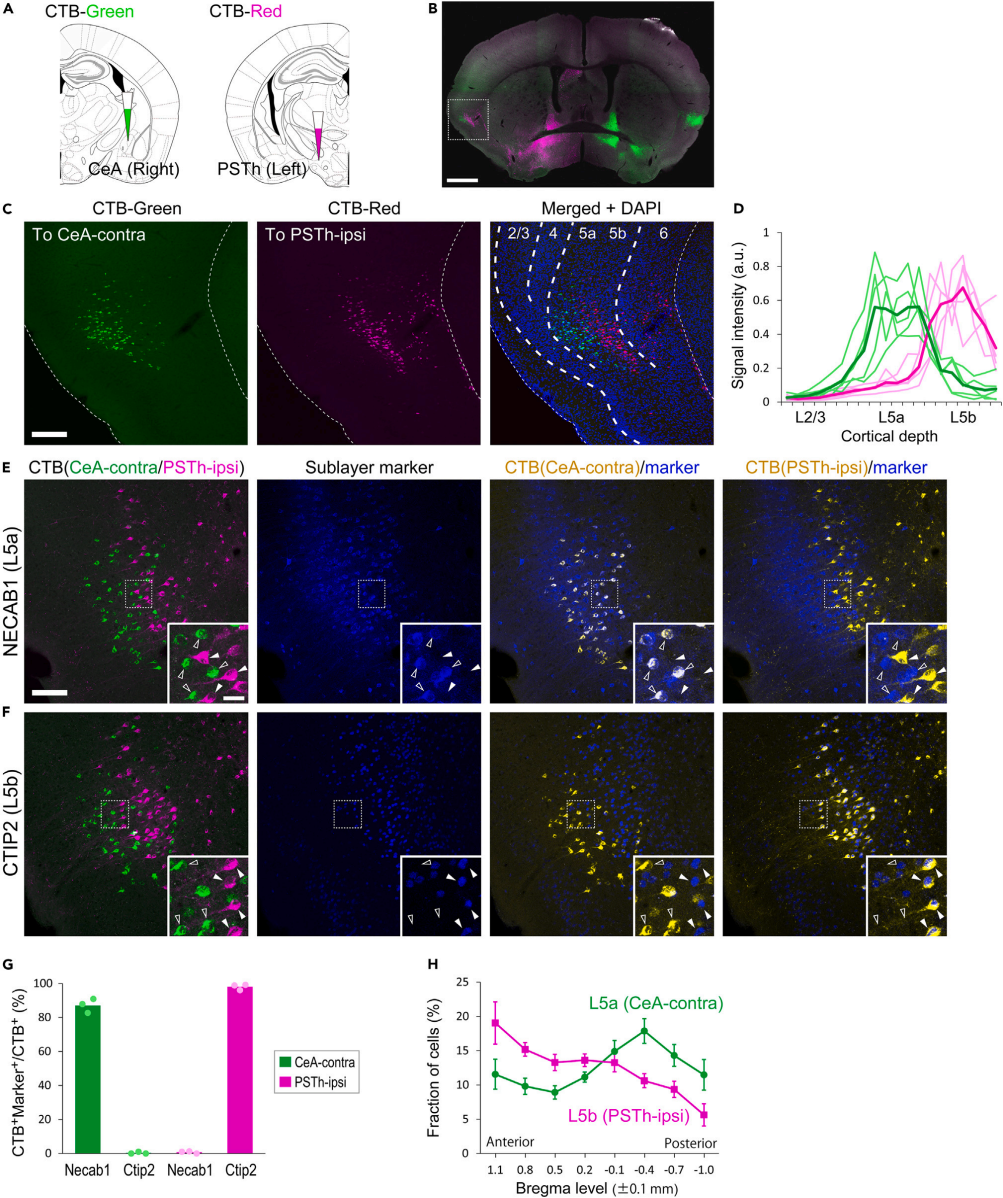
Sublaminar and regional distribution of anatomically distinct cell populations in L5 of the insula (A) Illustrations depicting dual-color CTB injections into the right CeA and left PSTh. (B) A typical example of the confocal image of a coronal section containing CTB-labeled cells in the insula. (C) Higher magnification views of the left insula (boxed region in B). (D) Relative fluorescence intensities (arbitrary unit) of CTB labeling across cortical depth in the DgI (L2/3 to L5b). Note that the density of labeled cells across cortical depth can be estimated by the averaged fluorescence because of the localized fluorescence to cell somas. The signal intensities of CTB-green-labeled cells (projecting to the CeA-contra) and CTB-red-labeled cells (projecting to the PSTh-ipsi) are shown in green and magenta, respectively. Light colors represent individual mice (n = 5 mice with dual CTB injections), and dark colors are the means. (E) Fluorescent images of a section containing CTB-labeled cells with immunostaining of NECAB1 (an L5a marker, blue). CeA-contra-projecting CTB-labeled cells (open arrowheads) but not PSTh-ipsi-projecting CTB-labeled cells (solid arrowheads) are immunopositive for NECAB1. (F) Fluorescent images of a section containing CTB-labeled cells with immunostaining of CTIP2 (an L5b marker, blue). PSTh-ipsi-projecting CTB-labeled cells (solid arrowheads) but not CeA-contra-projecting CTB-labeled cells (open arrowheads) are immunopositive for CTIP2. (G) The proportion of anatomically distinct CTB-labeled cell populations positive for the sublayer-specific molecular markers. Dots represent individual mice. Bars indicate the means (87.1% for NECAB1/CeA-contra; 0.33% for CTIP2/CeA-contra; 0.75% for NECAB1/PSTh-ipsi; 98.1% for CTIP2/PSTh-ipsi, n = 3 mice). (H) The fraction of CeA-contra-projecting CTB-labeled cells (L5a population, green) and PSTh-ipsi-projecting CTB-labeled cells (L5b population, magenta) at eight locations across the A-P axis of the DgI. The A-P level of the maximum proportion is 0.4 ± 0.1 mm posterior to Bregma for the L5a population (Dunnett’s test, p < 0.05 [versus −0.1 ± 0.1 mm]; p < 0.01 [versus −0.7 ± 0.1 mm]; p < 0.001 [versus 1.1, 0.8, 0.5, 0.2, −1.0 ± 0.1 mm], n = 6 mice) and 1.1 ± 0.1 mm anterior to Bregma for the L5b population (Dunnett’s test, p < 0.01 [versus 0.8 ± 0.1 mm]; p < 0.001 [versus 0.5, 0.2, −0.1, −0.4, −0.7, −1.0 ± 0.1 mm], n = 5 mice). Error bars represent standard deviations. Scale bars: 1 mm (B), 0.2 mm (C), 0.1 mm (E), 20 μm (inset in E). See also Figures S2 and S3.

We hypothesized that the axons with ipsilateral projections and those with contralateral projections originate from distinct neuronal populations of the insula. To test this hypothesis, using the contralateral CeL/CeC and ipsilateral PSTh as representative target regions for the lateralized projections, we performed dual injections of retrograde tracers cholera-toxin B subunit (CTB)-green and CTB-red into the right CeA–always covering at least a part of the CeL/CeC–and into the left posterior lateral hypothalamic area that includes the PSTh, respectively (Figures 2A and S2A). We found CTB-green- and CTB-red-labeled cells in the DgI in both hemispheres with distinct laminar distributions in the left hemisphere (Figure 2B, also see Figure S2B versus Figure S2C). In the left insula, cells retrogradely labeled from the contralateral (i.e., right) CeA (CeA-contra) were distributed primarily in L5a and sparsely in L5b (Figures 2C and 2D, green), whereas cells retrogradely labeled from the ipsilateral (i.e., left) PSTh (PSTh-ipsi) were predominantly located in L5b (Figures 2C and 2D, magenta) (also see Figure S2D). We found no double-labeled cells: 0 double-labeled cells out of 756 and 1,016 cells retrogradely labeled by CTB injections into the CeA-contra and PSTh-ipsi, respectively, analyzed in 10 coronal sections from five brains. This suggests that the contralateral CeL/CeC and ipsilateral PSTh (Figure 1) receive input from non-overlapping neuronal subpopulations in L5 of the DgI. To further characterize the sublaminar distribution of the subpopulations, we investigated the expression of molecular markers of L5 sublayers, namely, the N-terminal EF-hand calcium-binding protein 1 (NECAB1) for pyramidal neurons in L5a^34^ and the chicken ovalbumin upstream promoter transcription factor-interacting proteins 2 (CTIP2) for pyramidal tract-type neurons in L5b.^35^^,^^36^ Immunohistochemical analyses showed that these molecular expressions were clearly segregated within L5, whereas they were also found in superficial layers and L6 of the somatosensory cortex (Figure S2E). The immunostaining on CTB-labeled sections revealed that the majority of CeA-contra-projecting (87.1% on average, n = 3 mice) but virtually no PSTh-ipsi-projecting (0.7% on average, n = 3 mice) CTB-labeled cells were positive for NECAB1 (Figures 2E and 2G), whereas almost all PSTh-ipsi-projecting (98.1% on average, n = 3 mice) but virtually no CeA-contra-projecting (0.3% on average, n = 3 mice) CTB-labeled cells were positive for CTIP2 (Figures 2F and 2G). These results indicate that neurons contralaterally projecting to the CeL/CeC and those ipsilaterally projecting to the PSTh have distinct L5 sublayer-specific molecular profiles.

As the insula exhibits region specificity in subcortical projections,^2^^,^^3^^,^^27^ we examined the distribution of L5 subpopulations along the A-P axis. We found CeA-contra-projecting cells (the L5a population) in the entire DgI with the peak proportion of the cells at the middle of the posterior insula (0.4 ± 0.1 mm posterior to bregma) (Figure 2H, green). PSTh-ipsi-projecting cells (the L5b population) were found in the entire DgI as well; however, the proportion of the cells was highest at the most anterior part we analyzed (1.1 ± 0.1 mm anterior to bregma), which gradually decreased posteriorly (Figure 2H, magenta), suggesting an A-P gradient in the DgI. Both cell populations thus existed across the entire DgI with distinct A-P distributions.

Unlike the retrograde labeling from the contralateral CeA, retrograde labeling from the ipsilateral CeA resulted in cells distributed more broadly in the DgI layers (Figure S2C). Specifically, when the ipsilateral injection primarily covered the CeL/CeC, most retrogradely labeled cells were found in L5a and L2/3, and some of the L5a cells were double-labeled by the CTB injection into the contralateral CeA (Figures S3A and S3B). When the ipsilateral CTB injection mainly covered the CeM, most retrogradely labeled cells were found in L5b and L2/3, and only few cells were labeled in L5a (Figures S3A and S3C). These results suggest a layer-specific ipsilateral DgI input to the CeA: the CeL/CeC receives input from L5a and L2/3, whereas the CeM receives input from L5b and L2/3.

**Figure 3:**
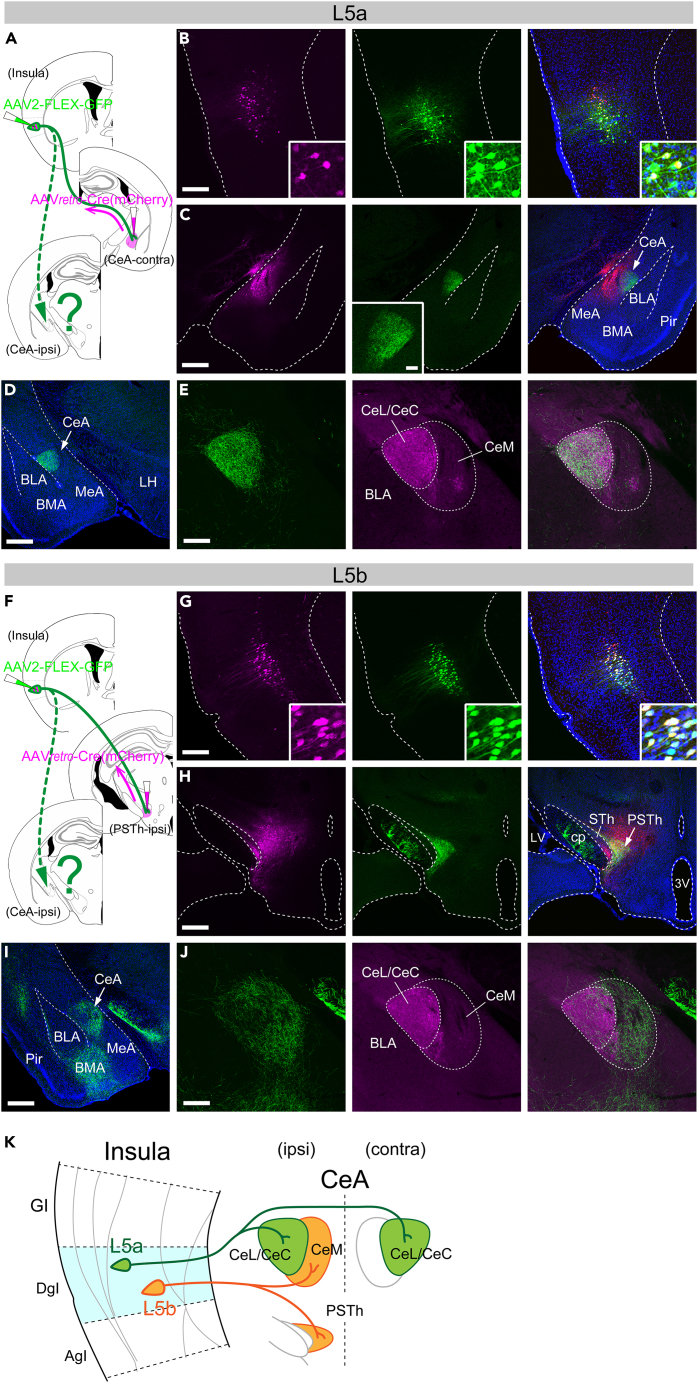
Differential organizations of axonal projections of DgI-L5 subpopulations to the CeA (A) Illustrations depicting AAV injections for Cre-induced GFP expression in the L5a population. Note that a retrograde AAV carrying Cre was injected into the CeA contralateral to the insula injected with an AAV carrying Cre-inducible GFP. (B) Fluorescent images of the AAV-injected site in the insula. Left: mCherry (a Cre expression reporter), middle: GFP, right: merged (also counterstained with DAPI, blue). (C) Fluorescent images of the AAV-injected site in the CeA. Left: mCherry (indicative of the AAV injection), middle: GFP (axon arbors restricted to the CeL/CeC, inset), right: merged (also with DAPI). (D) GFP-expressing axon arbors in the amygdala ipsilateral to the insula injected with the AAV vector. (E) A higher magnification view of the CeA ipsilateral to the insula with the AAV injection. Left: GFP (axon arbors restricted to the CeL/CeC), middle: anti-RGS14 immunostaining identifying the CeL/CeC but not CeM, right: merged. (F) Illustrations depicting AAV injections for Cre-induced GFP expression in the L5b population. (G) Fluorescent images of the AAV-injected site in the insula. Left: mCherry (Cre), middle: GFP, right: merged (also with DAPI). (H) Fluorescent images of the AAV-injected site in the ipsilateral hypothalamus. Left: mCherry (AAV injected site), middle: GFP (axon arbors restricted to the PSTh), right: merged (also with DAPI). (I) GFP-expressing axon arbors in the amygdala ipsilateral to the insula with the AAV injection. Labeled axons are found in the CeA and BMA. (J) A higher magnification view of the ipsilateral CeA. Left: GFP (axon arbors predominantly located in the CeM), middle: anti-RGS14 immunostaining, right: merged. Scale bars: 0.2 mm (B, E, G and J), 0.5 mm (C, D, H and I), 0.1 mm (inset in C). (K) Schematic diagram of the summary for CeA projections of DgI-L5 neuronal subpopulations. Abbreviations: 3V, third ventricle; BLA, basolateral amygdala; BMA, basomedial amygdala; cp, cerebral peduncle; LH, lateral hypothalamus; LV, lateral ventricle; MeA, medial amygdala; Pir, piriform cortex; STh, subthalamic nucleus. See also Figure S4.

To reveal the projection targets for axon collaterals of the CeA-contra-projecting L5a population and PSTh-ipsi-projecting L5b population, we expressed GFP selectively in each subpopulation and examined the distribution of the labeled axon arborizations. For GFP labeling of the L5a population, we combined the injection of a retrograde AAV carrying the Cre recombinase gene (AAVretro.EF1a.m.Cherry-ires-Cre)^37^ into the right CeA with the injection of an AAV encoding Cre-inducible GFP (AAV2.CAG.FLEX.GFP) into the left insula (contralateral to the Cre-virus-injected hemisphere) for GFP expression in the Cre-expressing neurons (Figures 3A and 3B). We observed GFP-expressing cell somas primarily in L5a of the left DgI, most (but not all) of which were also detectable for mCherry (the Cre expression indicator) (Figure 3B), and GFP-expressing axon arbors restricted to the CeL/CeC in the Cre-virus-injected contralateral CeA region which is marked by a mass of mCherry-expressing cells (Figure 3C). Like the contralateral projection, the ipsilateral GFP-labeled axon arbors in the amygdala were also confined to the CeL/CeC (Figures 3D, 3E, and S4A), indicating bilateral projections of the L5a subpopulation to the CeL/CeC. Moreover, we also found bilateral projections of GFP-labeled axons in the BNST, where the axon arbors were restricted to the STLD and juxtacapsular part of the BSTL (STLJ) (Figure S4C).

We took a similar strategy to label the axon collaterals of the L5b population. Specifically, we made injections of the AAVretro.EF1a.m.Cherry-ires-Cre into the PSTh and the AAV2.CAG.FLEX.GFP into the insula of the same hemisphere (Figure 3F). Besides GFP-expressing cell somas in L5b of the insula, we found, in the ipsilateral hemisphere, dense axon arbors in the PSTh (Figures 3G and 3H) and CeM and BMA of the amygdala, but only sparse terminals in the CeL/CeC (Figures 3I, 3J, and S4B). Thus, the organizations of ipsilateral projections originating from L5a and L5b subpopulations were largely complementary within the CeA, whereas the contralateral projection originated only from the L5a population and was confined to the CeL/CeC (Figure 3K). These results are compatible with our observation that the ipsilateral CTB injection biased to the CeM preferentially labeled the L5b cells, as well as L2/3 cells (Figure S3C), whereas the injection biased to the CeL/CeC preferentially labeled the L5a cells, as well as L2/3 cells (Figure S3B) in the DgI. In the BNST, we found that GFP-labeled axon arbors originating from the L5b population were localized in the STLP and STLV but not in the STLD/STLJ, only in the ipsilateral hemisphere (Figure S4D).

### Opposing modulatory roles of DgI L5 sublayers on appetitive drinking behavior

**Figure 4:**
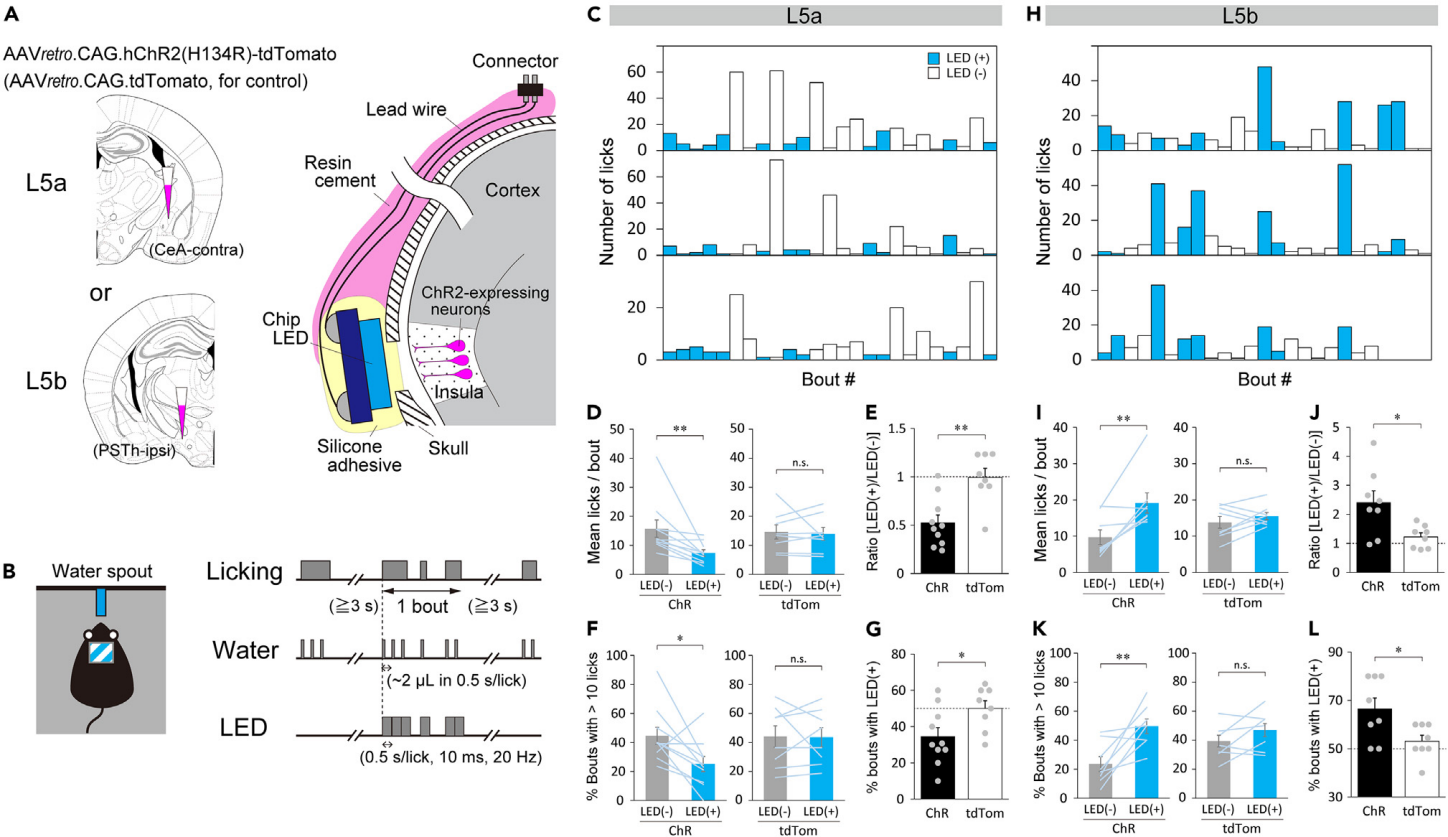
Opposite effects of the optogenetic activation of L5 sublayer neuronal populations on appetitive licking (A) Illustrations depicting AAV injections for retrograde ChR2 expression in the L5a or L5b populations (left) and the placement of a chip LED (right). (B) An illustration depicting a freely moving mouse with a single water spout (left) and the protocol for water delivery and illumination (right). Water (∼2 μL) is delivered when the mouse licks the spout. The illumination (10 ms, 20 Hz) is presented simultaneously at the beginning of water delivery only at random bouts. (C and H) Three typical examples of licking across a series of bouts with (blue) or without (white) illumination for mice expressing ChR2 in the L5a (C) and L5b (H) populations. Only a part of the session (25 bouts) is shown. (D and I) Comparisons of mean licks per bout between the bouts with (blue) and without (gray) LED illumination in ChR2-expressing (ChR) and control (tdTom) mice (L5a: 15.7 ± 3.0 for [LED-] versus 7.4 ± 1.1 for [LED+], Wilcoxon signed rank test, p < 0.01 for ChR, D-left; 14.6 ± 2.5 for [LED-] versus 13.9 ± 2.2 for [LED+], Wilcoxon signed rank test, p = 0.84 for tdTom, D-right; L5b: 9.7 ± 2.0 for [LED-] versus 19.2 ± 2.8 for [LED+], Paired t-test, p < 0.01 for ChR, I-left; 13.8 ± 1.6 for [LED-] versus 15.5 ± 1.0 for [LED+], Paired t-test, p = 0.30 for tdTom, I-right). (E and J) Ratio of the mean licks per bout with illumination relative to those without illumination (L5a: 0.53 ± 0.08 for ChR versus 0.99 ± 0.09 for tdTom, Student’s *t* test, p < 0.01, E; L5b: 2.41 ± 0.40 for ChR versus 1.22 ± 0.14 for tdTom, Welch’s *t* test, p = 0.022, J). (F and K) Proportion of long drinking bouts with >10 licks per bout in the session (L5a: 44.6 ± 5.9% for [LED-] versus 25.2 ± 5.2% for [LED+], Paired *t* test, p = 0.028 for ChR, F-left; 44.0 ± 7.4% for [LED-] versus 43.5 ± 6.6% for [LED+], Paired *t* test, p = 0.93 for tdTom, F-right; L5b: 23.6 ± 5.0% for [LED-] versus 49.5 ± 5.0% for [LED+], Paired *t* test, p < 0.01 for ChR, K-left; 39.3 ± 4.0% for [LED-] versus 46.9 ± 4.5% for [LED+], Paired *t* test, p = 0.12 for tdTom, K-right). (G and L) Percentage of bouts with illumination in the top 10 longest bouts in the session (L5a: 34.5 ± 4.9% for ChR versus 50.0 ± 4.3% for tdTom, Student’s *t* test, p = 0.034, G; L5b: 66.4 ± 4.6% for ChR versus 53.1 ± 2.5% for tdTom, Student’s *t* test, p = 0.022, L). Lines in D, F, I, and K and dots in E, G, J, and L represent individual mice (L5a: n = 10 for ChR, n = 8 for tdTom; L5b: n = 8 for ChR, n = 8 for tdTom). Dotted lines in E, G, J, and L indicate the chance level. ∗p < 0.05, ∗∗p < 0.01. See also Figure S5.

The anatomical findings described above strongly suggest a functional difference between the two subpopulations of L5 neurons in the DgI. Recent studies have reported that the insula bidirectionally modulates drinking behavior in a region-specific manner,^1^^,^^2^ whereas the CeL bidirectionally regulates feeding in a neuronal subtype-specific fashion.^38^^,^^39^^,^^40^^,^^41^ In addition, the CeC and CeM were reported to play opposing roles in appetitive behaviors,^38^ and the PSTh was shown to participate in the processing of palatability^32^ or, on the contrary, the suppression of feeding.^41^^,^^42^^,^^43^^,^^44^^,^^45^ These findings prompted us to ask whether the sublayers of the DgI L5 play distinct roles in the modulation of appetitive behavior. Therefore, we examined the effect of optogenetic activation of each sublaminar neuronal population on drinking behavior in thirsty mice, a model of appetitive behavior. We expressed Channelrhodopsin-2 (ChR2(H134R))^46^^,^^47^ fused with tdTomato in either the L5a population (bilaterally projecting to the extended amygdala) or the L5b population (ipsilaterally projecting to the amygdala and brainstem) using a retrograde AAV (AAVretro.CAG.hChR2-H134R-tdTomato). We made a unilateral injection of the virus: the contralateral CeA injection for the L5a population activation or ipsilateral PSTh injection for the L5b population activation (Figure 4A), and illuminated insula of only one hemisphere that exhibited sublayer-specific ChR2 expression (Figures S5A and S5C), because bilateral injections of the ChR2 virus into the CeA can cause ChR2 expression in multiple DgI layers (i.e., L2/3, L5a, and L5b) that contain both ipsilaterally and contralaterally projecting neuronal populations (Figure S3) and make it practically impossible to optogenetically activate the L5a or L5b populations selectively. Because of no anatomical difference between the left and right insulae (Figure S1), we applied the optical stimulation to the left insula in all the following optogenetic experiments. To optically stimulate ChR2-expressing neurons, a chip light-emitted diode (LED) was embedded over a small cranial window above the middle-posterior insula and was wirelessly controlled to deliver blue light to the cortex with the dura mater intact (Figure 4A). This method was thus noninvasive to the cortex. The effectiveness of optogenetic stimulation was confirmed by detecting c-Fos expression in the DgI, induced by repetitive LED illuminations at the end of all behavioral experiments in each mouse: c-Fos immunoreactivity was primarily found in the ChR2-expressing layer of the stimulated side of the DgI (Figures S5A and S5C), and the number of c-Fos positive cells in the stimulated side was significantly higher than in the non-stimulated side in mice expressing ChR2, but not in control mice expressing only tdTomato (Figures S5B and S5D), regardless of the callosal connections to the opposite side of the insula (Figure 1E).

We first tested whether and how licking of a water spout was influenced by the activation of each neuronal population in freely moving mice. To execute this, we presented the LED light during licking in random drinking bouts (a bout is a licking cluster sandwiched by ≥ 3 s non-licking periods) (Figure 4B). In mice expressing ChR2 in the L5a population, we found that the number of licks was markedly smaller in many bouts with LED illumination than in the bouts without illumination (Figure 4C, also see Video S1). The mean number of licks per bout was significantly smaller in the bouts with illumination compared with those without illumination (Figure 4D-left). Because the mice continued licking to some extent during illumination (Figure 4D-left, 7.4 ± 1.1 licks per bout with illumination, Video S1), it is unlikely that the illumination directly interrupted bodily movements for drinking or caused seizures. When tested in control mice whose L5a population expressed only tdTomato (Figure 4A), the mean number of licks per bout showed no change in the bouts with LED illumination compared with those without illumination (Figure 4D-right), indicating that the illumination per se has no effect on drinking behavior. The mean number of licks per bout was reduced approximately by half on average by the illumination in the mice with ChR2 expression (Figure 4E). The suppression of licking by opto-activation of the L5a population was also manifested in bouts with a larger number of licks. The maximum number of licks per bout was decreased by less than half on average by the illumination in the ChR2-expressing mice (Figure S5E). Further, the proportion of long drinking bouts (with more than 10 licks per bout) in the session was significantly lower for the bouts with illumination compared with those without illumination, whereas no difference was found in the control mice (Figure 4F). In addition, the percentage of bouts with illumination in the top 10 longest bouts (with the highest number of licks) was also significantly lower in the ChR2-expressing mice than in control mice (Figure 4G). In contrast to the mice whose L5a population expressed ChR2, mice whose L5b population expressed ChR2 showed markedly higher number of licks per bout in the bouts with illumination compared with those without illumination (Figures 4H and 4I, also see Video S2). The mean number of licks per bout was increased more than twice on average by the illumination in the ChR2-expressing mice (Figure 4J). The facilitation of licking by opto-activation of the L5b population was also manifested in the maximum number of licks per bout (Figure S5F), the proportion of long drinking bouts (Figure 4K), and the percentage of bouts with illumination in the top 10 longest bouts (Figure 4L). Of interest, the total number of drinking bouts across the session was not significantly altered by the opto-activation of both L5a and L5b populations (Figure S5G), suggesting the absence of a persistent effect after illumination.

Video S1. A typical example of water spout licking without versus with opto-activation for a mouse whose L5a population expresses ChR2, related to Figure 4

Video S2. A typical example of water spout licking without versus with opto-activation for a mouse whose L5b population expresses ChR2, related to Figure 4

The opto-activation of the posterior insula and insula-CeA circuits in a previous study induced anxiety-related behavior (3), which could lead to the suppression of drinking behavior. To test whether the opto-activation of the L5a population induces anxiety, we analyzed exploratory behavior during illumination in an open field (OF) (Figure S6A)^48^ and an elevated plus maze (EPM) (Figure S6G).^49^ The opto-activation neither altered general locomotor activities (i.e. the distance traveled, Figures S6B and S6C) nor decreased time spent in the center of the OF (Figures S6D and S6F). We also observed no statistically significant reduction of exploratory activities in the open arms of the EPM although some mice exhibited virtually no activity in the open arms (Figures S6H and S6I). It is thus unlikely that the opto-activation of the L5a population per se generates anxiety or fear. Similarly, the opto-activation of the L5b population neither affected general locomotor activities (Figures S6B and S6C) and exploratory behavior in the OF (Figures S6E and S6F) nor increased exploratory activity in the open arms of the EPM (Figures S6H and S6I), suggesting no anxiolytic effect of the L5b opto-activation.

**Figure 5:**
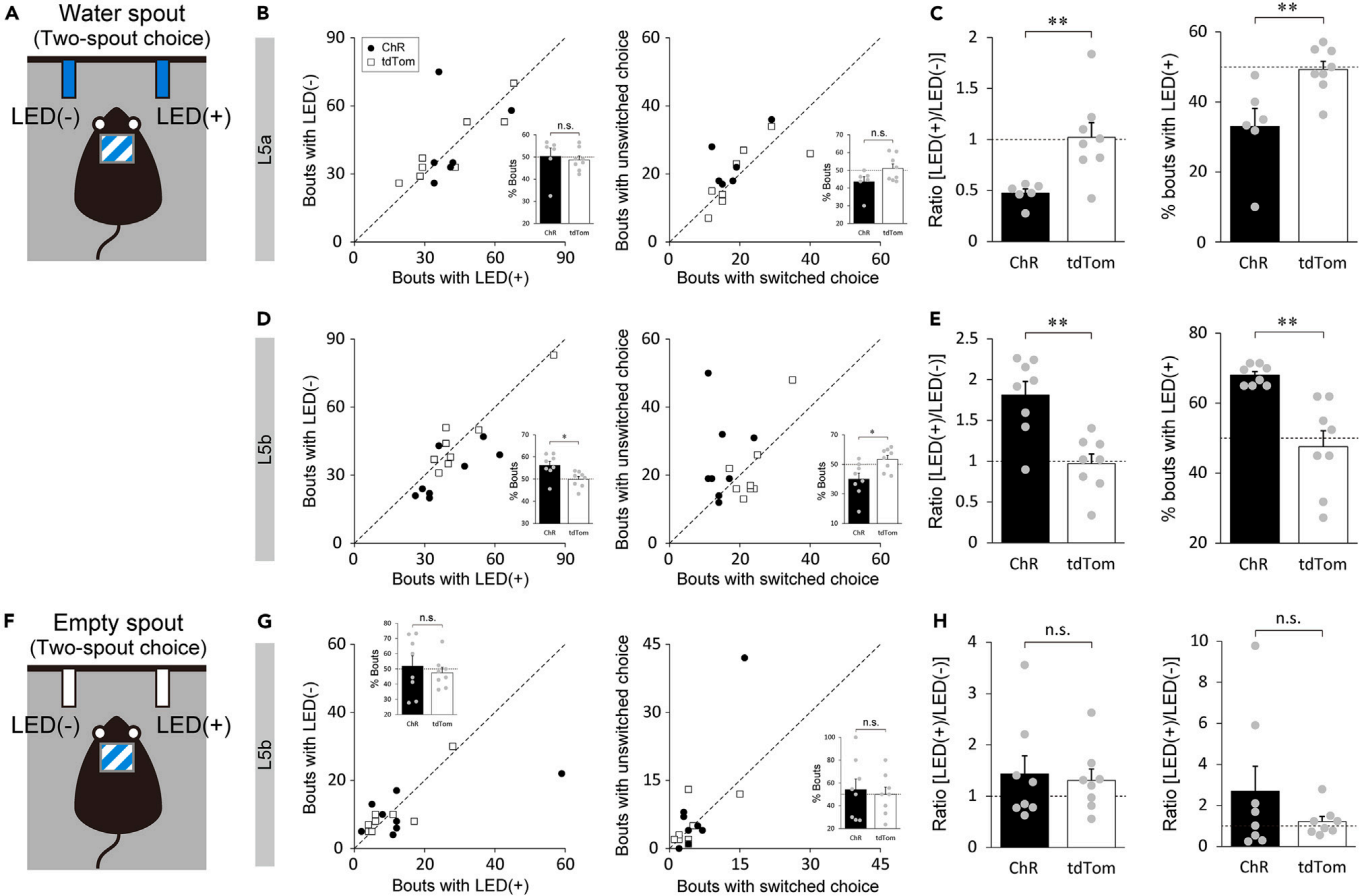
Lack of emotional valence induced by optogenetic activation of L5 subpopulations (A) An illustration depicting a freely moving mouse in a two-spout choice test with one water spout paired with LED illumination and the other unpaired with LED illumination. Note that the two spouts deliver the same amount of water per lick. (B and D) Left: the number of bouts in which the mouse chose the spout with versus without illumination for mice expressing ChR2 (black circles) or tdTomato (white squares) in the L5a population (ChR: 42.3 ± 5.1 for [LED+] versus 43.7 ± 7.7 for [LED-], Wilcoxon signed rank test, p = 0.56; tdTom: 41.0 ± 6.3 for [LED+] versus 41.8 ± 5.4 for [LED-], Paired *t* test, p = 0.78, B) and L5b population (ChR: 39.9 ± 4.7 for [LED+] versus 31.3 ± 3.8 for [LED-], Paired *t* test, p = 0.024; tdTom: 45.9 ± 5.9 for [LED+] versus 46.1 ± 5.8 for [LED-], Paired *t* test, p = 0.91, D). Inset bar graphs represent the percentage of bouts paired with illumination for (L5a: 50.3 ± 3.7% for ChR versus 48.8 ± 1.7% for tdTom, Wilcoxon rank-sum test, p = 0.30, B; L5b: 56.2 ± 1.8% for ChR versus 49.9 ± 1.3% for tdTom, Student’s *t* test, p = 0.013, D). Right: the number of bouts in which the mouse switched versus unswitched choice next to the bouts with illumination (ChR: 17.8 ± 2.5 for switched choice versus 23.2 ± 3.1 for unswitched choice, Paired *t* test, p = 0.071; tdTom: 20.3 ± 3.5 for switched choice versus 19.8 ± 3.2 for unswitched choice, Paired *t* test, p = 0.84 for the L5a population, B; ChR: 14.8 ± 1.5 for switched choice versus 24.5 ± 4.4 for unswitched choice, Paired *t* test, p = 0.047; tdTom: 23.4 ± 1.9 for switched choice versus 21.8 ± 4.0 for unswitched choice, Paired *t* test, p = 0.56 for the L5b population, D). Inset bar graphs represent the percentage of bouts paired with illumination for (L5a: 43.6 ± 2.9% for ChR versus 51.1 ± 2.6% for tdTom, Wilcoxon rank-sum test, p = 0.24, B; L5b: 40.0 ± 4.0% for ChR versus 53.4 ± 2.7% for tdTom, Student’s *t* test, p = 0.015, D). (C and E) Left: the ratio of the mean licks per bout with illumination relative to those without illumination for mice expressing ChR2 (black) or tdTomato (white) in the L5a population (0.47 ± 0.04 for ChR versus 1.02 ± 0.14 for tdTom, Welch’s *t* test, p < 0.01, C) and L5b population (1.81 ± 0.17 for ChR versus 0.97 ± 0.12 for tdTom, Student’s *t* test, p < 0.01, E). Right: the percentage of bouts with illumination in the top 10 longest bouts in the sessions (L5a: 33.0 ± 5.2% for ChR versus 49.3 ± 2.4% for tdTom, Student’s *t* test, p < 0.01, C; L5b: 68.0 ± 1.0% for ChR versus 47.5 ± 4.6% for tdTom, Welch’s *t* test, p < 0.01, E). Gray dots represent individual mice (n = 6 for ChR, n = 8 for tdTom in B and C; n = 8 for ChR, n = 8 for tdTom in D and E). (F) An illustration depicting a freely moving mouse in a two-spout choice test with one spout paired with and the other unpaired with LED illumination. Note that both spouts were devoid of water. (G) Left: the number of bouts in which the mouse chose the spout with versus without illumination for mice whose L5b population expressed ChR2 (black circles) or tdTomato (white squares) (ChR: 15.1 ± 6.4 for [LED+] versus 10.6 ± 2.2 for [LED-], Wilcoxon signed rank test, p = 0.64; tdTom: 10.1 ± 3.0 for [LED+] versus 10.4 ± 2.9 for [LED-], Wilcoxon signed rank test, p = 0.42). Inset bar graphs represent the percentage of bouts paired with illumination for (51.9 ± 6.6% for ChR versus 47.5 ± 3.6% for tdTom, Student’s *t* test, p = 0.57). Right: the number of bouts in which the mouse switched versus unswitched choice next to the bouts with illumination (ChR: 5.6 ± 1.6 for switched choice versus 8.9 ± 4.8 for unswitched choice, Wilcoxon signed rank test, p = 0.53; tdTom: 4.6 ± 1.6 for switched choice versus 5.0 ± 1.7 for unswitched choice, Wilcoxon signed rank test, p = 0.88). Inset bar graphs represent the percentage of bouts paired with illumination for (54.1 ± 9.3% for ChR versus 49.9 ± 6.4% for tdTom, Student’s *t* test, p = 0.71). (H) Left: the ratio of the mean licks per bout with illumination relative to those without illumination for mice whose L5b population expressed ChR2 or tdTomato (1.43 ± 0.35 for ChR versus 1.31 ± 0.22 for tdTom, Wilcoxon rank-sum test, p = 0.88). Right: the ratio of the total number of licks in the session (2.70 ± 1.20 for ChR versus 1.21 ± 0.25 for tdTom, Wilcoxon rank-sum test, p = 0.88). Gray dots represent individual mice (n = 8 for ChR, n = 8 for tdTom). Dotted lines in B-E, G, and H indicate the chance level. See also Figure S7.

We further asked whether the behavioral changes induced by opto-activation of the L5 neuron subpopulations are either caused by aversive and hedonic emotional responses to the spout with illumination that led to the suppression and facilitation of licking, respectively, or are attributed only to the modulation of the motivational aspect of licking without emotional change. To address this question, we performed a two-spout choice test in which mice can freely choose either the spout paired with illumination or the unpaired one, both spouts of which deliver equal amount of water per lick (Figure 5A). We reasoned that if opto-activation during licking induces aversive emotional valence, the mice would show preference toward the spout unpaired with illumination and that if opto-activation induces appetitive emotional valence, the mice would prefer the spout paired with illumination. This argument was supported by our validation of the two-spout choice test using intact B6 mice with quinine and sucrose as innate aversive and appetitive tastants, respectively. The mice preferred the water spout over the quinine spout in the water-quinine choice test and the sucrose spout over the water spout in the water-sucrose choice test while showing no preference between the two spouts delivering water (Figure S7A). The mice also exhibited more frequent spout switching when they licked the quinine spout in the water-quinine choice test and less frequent spout switching when they licked the sucrose spout in the water-sucrose choice test (Figure S7B). Testing the mice, whose L5a population expressed ChR2, with both spouts delivering water revealed that opto-activation reduced the mean and maximum number of licks per bout and the proportion of long bouts (Figures 5C and S7C–S7E), in line with the single-spout test results (Figure 4D–4G and S5E), but affected neither the choice of spout nor spout switching (Figure 5B). These results suggest that opto-activation of the L5a population has no effect on negative emotional valence but suppresses the motivation of licking.

Activation of the L5b population revealed that a portion of mice showed a higher frequency of choice of the spout with illumination compared with of the spout without illumination and less frequent spout switching when they chose the spout with illumination. On average, the number of choices of the illumination-paired spout and that of unswitched choices next to the bouts with illumination were slightly but significantly higher than that of the illumination-unpaired spout and that of switched choice, respectively (Figure 5D). In addition, a marked increase was noted in the mean and maximum number of licks per bout with illumination and the proportion of long drinking bouts with illumination in ChR2-expressing mice (Figures 5E and S7F–S7H). To test whether opto-activation of the L5b population is sufficient to induce a positive valence and elicit the preference for the spout with illumination, using the same group of mice (non-deprived of water before the test), we further performed another two-spout choice test in which one of the two spouts was paired with illumination, but neither delivered water (Figure 5F). The results showed that the mice did not frequently lick the spout even paired with illumination. There was no significant difference in the number of choices between the spouts with and without illumination and between switched and unswitched choices, although one of the eight ChR2-expressing mice tested exhibited exceptionally a remarkable preference for the spout with illumination (Figure 5G). Similarly, the mean number of licks per bout and the total number of licks in the session were not significantly increased by illumination in ChR2-expressing mice compared with control mice (Figure 5H). Thus, it is unlikely that the activation of the L5b population per se induces emotional valence, although it may enhance the incentive to drink water.

## Discussion

**Figure 6:**
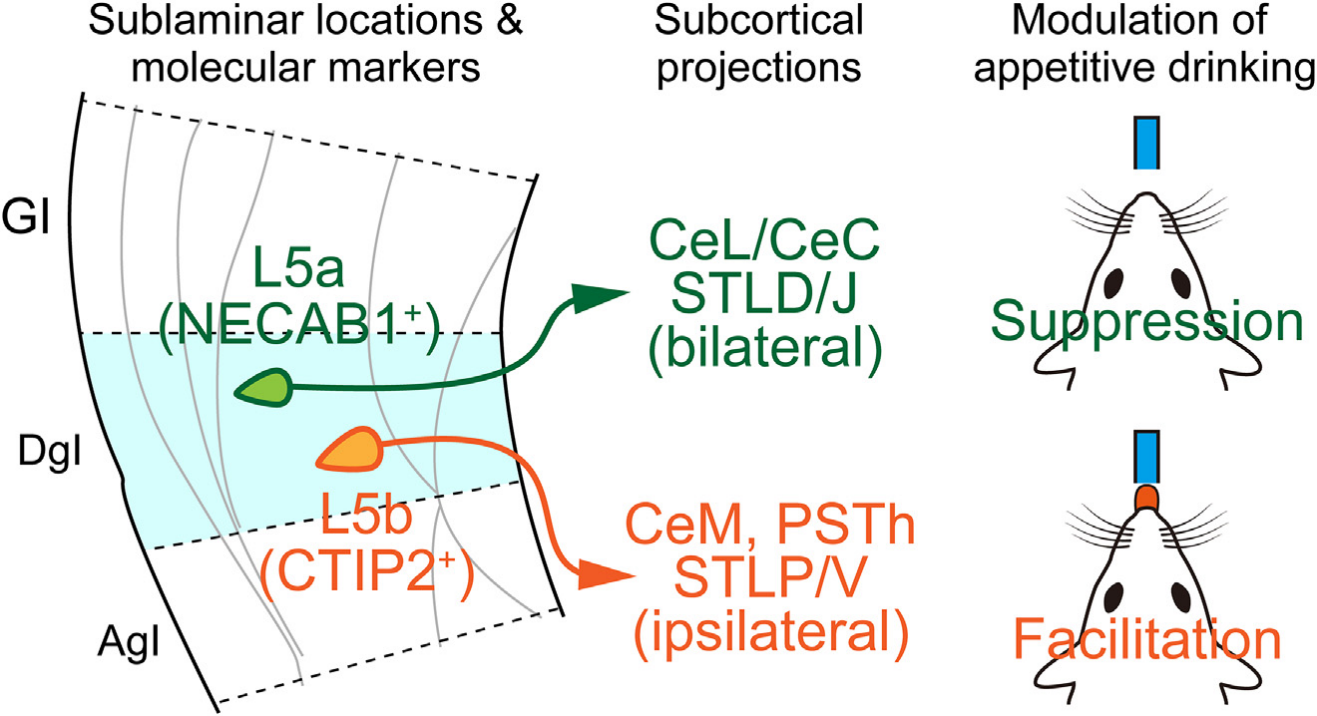
Summary of the study Molecular, anatomical, and functional characteristics of sublaminar neuronal populations in L5 of the DgI.

Functional differences among anatomically distinct L5 neuronal subpopulations have been reported in sensory,^50^^,^^51^ frontal/motor,^52^^,^^53^ prefrontal,^54^^,^^55^ and anterior cingulate^56^ cortices of rodents. The anatomical and functional heterogeneity in L5 neurons of the insula, however, remains poorly understood, despite the wide variety of subcortical efferents of the insula and their difference between A-P subregions.^2^^,^^21^^,^^23^^,^^27^ In this study, by taking advantage of the hemispheric difference in the subcortical projections, we succeeded in dissecting L5 neurons of the DgI into two subpopulations with distinct sublaminar distributions, molecular expressions, axonal targets in the extended amygdala and hypothalamus, and modulatory effects on appetitive drinking (Figure 6).

### L5a population as a suppressor of appetitive behavior

Previous optogenetic studies have suggested that the insula-CeA circuit serves to suppress feeding and drinking behaviors.^2^^,^^3^^,^^8^^,^^16^ Because ChR2 was expressed in the insula ipsilateral to the illuminated side in all of the studies, it is plausible that the illumination above ChR2-expressing axon terminals in the CeA simultaneously activated the axons originating from multiple layers of the insula, including the L5a subpopulation (projecting to the CeL/CeC) and L5b subpopulation (projecting to the CeM), which may contribute opposingly to appetitive drinking, based on our results (Figure 4). In this sense, the L5a to CeL/CeC pathway is likely a critical component of insula-CeA circuits involved in the suppression of appetitive behavior. Possibly, the L5a to CeL/CeC pathway was activated more effectively than the L5b to CeM pathway by optogenetic techniques used in the previous studies. It is also possible that the activity of the L5a-CeL/CeC circuit has more impact on appetitive behavior by default when both L5a and L5b pathways are activated simultaneously. Nevertheless, it is worth noting that the net effect of insula L5-CeA circuit activities on appetitive behaviors depends on how strong each of the sublayer axons is synaptically connected with functionally diverse subpopulations of CeA neurons and on which of the connections is potentiated by experiences.^16^

Our evidence is against the interpretation of licking suppression as an aversive response because opto-activation of the L5a population induced no avoidance against the water spout paired with illumination in our two-spout choice test. This result contradicts that from previous studies showing that optogenetic activation of the insula^1^^,^^3^^,^^4^ or insula-CeA circuits^2^^,^^8^ induced an avoidance behavior in a place preference test, although no anxiogenic effect was observed in an open-field test with opto-activation of insula-CeA circuits^16^ in accordance with the present study (Figures S6B–S6D and S6F). It is possible that the L5a population selectively serves to attenuate the incentive salience in appetitive behavior, and other neuronal populations and their circuits are necessary for the generation of negative emotional valence. One may wonder whether the licking suppression by opto-activation of the L5a population was because of a direct inhibition of bodily movements for drinking rather than the attenuation of the motivation of drinking. Our observation that licking behavior lasted to some extent during the illumination, despite the number of licks was smaller than that in the bouts without illumination, argues against this possibility (Figures 4C–4G). Moreover, if the mice had been interrupted from drinking by illumination in the two-spout choice test, they should have preferred the spout without illumination; but the result showed no preference for the spout (Figure 5B). It is thus most likely that the opto-activation suppressed the motivational aspect of drinking behavior, possibly acting as a teaching signal for transient satiety or other behavioral needs with a higher priority.

### Possible neural pathways of L5b involved in the facilitation of appetitive behavior

In contrast to the suppressive effect by the L5a population, we found the facilitatory effect of the L5b opto-activation on drinking behavior (Figures 4H–4L) but not on licking behavior per se (Figures 5G and 5H), suggesting that the L5b population may contribute to the enhancement of incentive salience by recruiting ‘wanting’ neural circuits.^57^ This notion is in line with the functional properties of the CeM, the amygdala target region of the L5b population, where essentially all neuron subtypes promote appetitive behaviors.^38^ However, the role of the PSTh, another target of the L5b population, is currently controversial; it responds to palatable food^32^ but is activated in neophobic^45^ and anorexigenic^43^^,^^45^ situations and is also activated by a conditioned aversive taste although not by bitter taste.^42^ The latter results agree with a study in which optogenetic activation of a PSTh neuronal population projecting to the PVT resulted in feeding suppression.^44^ The controversy in the role of PSTh could be attributable to its neuronal diversity; the PSTh contains cell populations expressing preprotachykinin-1,^32^^,^^45^^,^^58^ calbindin,^32^^,^^59^ corticotropin-releasing hormone,^43^ or vesicular glutamate transporter 2.^44^ Therefore, it is crucial to identify the neuronal population of the PSTh that is specifically innervated by the L5b population of the insula. Besides the CeM and PSTh, the L5b population of the insula characterized in our study has multiple subcortical targets, such as the pons and medulla. Insula L5b population is, therefore, reminiscent of pyramidal tract-type neurons in the neocortex.^60^^,^^61^^,^^62^ This idea is supported by the expression of the Ctip2 gene in both neuron types^35^^,^^63^ (Figures 2F and 2G). How the down-stream targets of the insula are involved is not clear at this time, and the involvement of none of the targets can be excluded at this time. How the divergent projections of the L5b population contribute to the facilitation of appetitive behavior also needs to be unraveled in future studies with an opto-stimulation of axon terminals in the target regions. The present study demonstrated that unilateral (left) opto-activation of insula L5 neuronal populations was sufficient for behavioral modulations (Figure 4). Although the effect of the right or bilateral activations of these populations remains to be clarified in the future, subcortical input from both hemispheres of the insula may have the same effect on behavioral modulation based on the anatomical homogeneity (Figure S1). Our results by no means suggest lateralization of insula function to one side.

Like the activation of the L5a population, opto-activation of the L5b population in the middle-posterior DgI had no effect on the induction of emotional behaviors (Figures 5G, 5H, S6B, S6C, S6E, and S6F). However, because the posterior insula has been reported to have a hedonic ‘liking’ hot spot,^57^^,^^64^ other layers or subregions (e.g., GI and AgI) of the posterior insula might be linked to neural circuits involved in positive emotions.

### A perspective of the insula’s role in taste quality-independent modulation of feeding/drinking

Our result of the anterior-low/posterior-high distribution of the L5a population projecting to the CeL/CeC (Figure 2H) is in line with the previous studies demonstrating an A-P region difference in the insula-mediated modulation of drinking^1^ and neural circuits of the anterior insula-BLA and posterior insula-CeA involved in the facilitation and suppression, respectively.^2^ Our finding of the distribution of the L5b population projecting to the PSTh/CeM with an anterior-high/posterior-low gradient (Figure 2H) further suggests its participation in the facilitatory role of the anterior insula^1^ in concert with the neuronal populations connecting to the BLA.^2^ On the other hand, despite the difference in distributions along the A-P axis, both L5 subpopulations in the DgI are found along the *entire* axis. This result raises the possibility that the insula exerts the suppressive and facilitative effects in an A-P axis-independent fashion. Although taste coding in the gustatory cortex is still controversial, L5 sublayers of the DgI could be available for both “topographical taste coding”^17^^,^^65^ and “sparse or distributed coding”.^18^^,^^19^ In either case, the sublayer-specific neural circuitries are potential substrates for the regulation of adaptive feeding/drinking behavior in response to a variety of tastes rather than for taste quality-specific hard-wired modulations. An issue to be addressed in the future is whether the activation of the L5b subpopulation, by enhancing the motivation of feeding/drinking, can overcome aversive tastes, whereas it has been reported that optogenetic activation of the posterior insula-CeA circuit suppresses the intake of an appetitive taste.^2^^,^^3^

In addition to by taste qualities, motivation for feeding/drinking can be affected by taste-independent contexts, including homeostatic, emotional, and external circumstances, although all of which can be associated with any type of taste by learning and lead to anticipatory behaviors.^5^^,^^6^^,^^7^^,^^8^^,^^9^^,^^10^^,^^11^^,^^12^ Thus, clarifying the properties of sensory and contextual inputs to each L5 sublayer of the insula and the necessity of the neuronal populations for behavioral decisions driven by those inputs is also a critical issue for future studies. In the present study, the L5 opto-stimulation was limited to water drinking epochs and also to an experimental context and, therefore, it remains to be addressed whether a sustained activation of the L5 sublayer can alter daily water consumption, which might be crucial for understanding the insula’s role in anorexia/hyperorexia. In addition, although we observed a statistically undetectable effect of the L5 opto-activation on exploratory activities in the open arms in our EPM setting (Figures S6H and S6I), it remains possible that the insula L5 has a role in the efficiency control of an emotional impact on motivated behavior. It is thus intriguing to address whether the insula L5 sublayers contribute to the modulation of not solely feeding/drinking behavior but rather emotion-related motivational behaviors in general.

### Limitations of the study

Our anatomical study clearly demonstrated that L5 sublayers of the mouse dysgranular insula have distinct subcortical projections. It remains to be clarified whether the projection targets receive topographical input from the insula. In the present study, we unilaterally illuminated only the left insula for selective opto-activation of an L5 sublayer population where ChR2 was expressed by the retrograde AAV infection. Whether opto-activation of the right or bilateral insulae has the same effect on appetitive drinking behavior remains to be investigated; such studies will clarify the laterality of the insula’s function. In addition, we assessed the function of the middle-posterior insula only in the current study; the roles of L5 sublayers in the anterior insula remain to be unveiled. Finally, our opto-activation study suggests modulatory roles for L5 sublayers in appetitive drinking, but how neurons of each sublayer are engaged in feeding/drinking behaviors is an important issue to be addressed.

## STAR★Methods

### Key resources table

**Table:** 

REAGENT or RESOURCE	SOURCE	IDENTIFIER
**Antibodies**		

Anti-c-Fos, rabbit polyclonal	Sigma-Aldrich	Cat# F7799; RRID: AB_259739
Anti-CTIP2, rat monoclonal [25B6]	Abcam	Cat# ab18465; RRID: AB_2064130
Anti-FOXP2, rabbit polyclonal	Abcam	Cat# ab16046; RRID: AB_2107107
Anti-FOXP2, mouse monoclonal	Atlas Antibodies	Cat# AMAb91361; RRID: AB_2716651
Anti-GFP, rat monoclonal [GF090R]	Nacalai Tesque Inc., Kyoto Japan	Cat# 04404-84; RRID: AB_10013361
Anti-NECAB1, rabbit polyclonal	Atlas Antibodies	Cat# HPA023629; RRID: AB_1848014
Anti-PKCδ, mouse monoclonal	BD Biosciences	Cat# 610398; RRID: AB_397781
Anti-RGS14, mouse monoclonal [NeuroMab clone N133/21]	NeuroMab, Davis CA	Cat# 75-170; RRID: AB_2179931
Donkey anti-Rat IgG (H+L) Highly Cross-Adsorbed Secondary Antibody, Alexa Fluor 488	Thermo Fisher Scientific	Cat# A-21208; RRID: AB_2535794
Donkey Anti-Mouse IgG H&L (Alexa Fluor® 647) preadsorbed	Abcam	Cat# ab150111; RRID: AB_2890625
Donkey Anti-Rabbit IgG H&L (Alexa Fluor® 647) preadsorbed	Abcam	Cat# ab150063; RRID: AB_2687541
Donkey anti-Rabbit IgG (H+L) Highly Cross-Adsorbed Secondary Antibody, Alexa Fluor Plus 647	Thermo Fisher Scientific	Cat# A32795; RRID: AB_2762835
Donkey anti-Rabbit IgG (H+L) Highly Cross-Adsorbed Secondary Antibody, Alexa Fluor 488	Thermo Fisher Scientific	Cat# A-21206; RRID: AB_2535792

**Bacterial and virus strains**		

rAAV2-CAG-GFP	UNC Vector Core (Edward Boyden)	N/A
rAAV2-CAG-FLEX-GFP	UNC Vector Core (Edward Boyden)	N/A
pAAV-Ef1a-mCherry-IRES-Cre (AAV Retrograde)	Karl Deisseroth; Fenno et al.^66^	Addgene; 55632-AAVrg
AAV-CAG-hChR2-H134R-tdTomato (AAV Retrograde)	Karel Svoboda; Mao et al.^67^	Addgene; 28017-AAVrg
pAAV-CAG-tdTomato (codon diversified) (AAV Retrograde)	Edward Boyden	Addgene; 59462-AAVrg

**Biological samples**		

C57BL/6JJmsSlc	Japan SLC, Shizuoka Japan	N/A

**Chemicals, peptides, and recombinant proteins**		

Cholera Toxin Subunit B (Recombinant), Alexa Fluor™ 488 Conjugate	Thermo Fisher Scientific	Cat#C22841
Cholera Toxin Subunit B (Recombinant), Alexa Fluor™ 555 Conjugate	Thermo Fisher Scientific	Cat#C22843

**Software and algorithms**		

ImageJ 1.52a	NIH	https://imagej.nih.gov/ij/
Arduino IDE 1.8.12	Arduino.cc	https://www.arduino.cc/en/software
R version 4.0.4	The R Foundation	https://www.r-project.org/
ANY-maze v6.17 and v7.16	Stoelting Co.	https://www.any-maze.com/

**Other**		

Chip LED (470 nm)	OptoSupply Ltd, N.T., Hong Kong	Cat#OSBL1608C1A
Teleopto receiver	Bio Research Center Co., Ltd., Aichi Japan	Cat#TeleR-1-P
Teleopto infrared emitter	Bio Research Center Co., Ltd., Aichi Japan	Cat#TeleEmitter
Teleopto controller	Bio Research Center Co., Ltd., Aichi Japan	Cat#TeleRemocon
Arduino UNO Rev3	Arduino, Somerville, MA	Cat#A000066

### Resource availability

#### Lead contact

Further information and requests for resources and reagents should be directed to and will be fulfilled by the lead contact, Makoto Takemoto (takemoto@kumamoto-u.ac.jp).

#### Materials availability

This study did not generate new unique reagents.

### Experimental model and study participant details

Male C57BL/6J (B6) mice (Japan SLC, Shizuoka, Japan) were used for all experiments. At the time of the injection experiments (see below), the mice were 6–10 weeks old for anatomical studies and were 5–7 weeks old for optogenetic studies. All experiments were approved by the Committee for Animal Experiments of Kumamoto University and performed in accordance with the Guidelines for Use of Animals in Experiments of Kumamoto University.

### Method details

#### Surgery

Mice were anesthetized with a mixture of ketamine (Ketamine injection, Fujita Pharmaceutical, Tokyo) and xylazine (Selactar, Bayer Yakuhin, Tokyo) (ketamine 80 mg/kg, xylazine 8 mg/kg) during surgery. For injections of CTB or viruses into subcortical regions (i.e., the CeA and PSTh), mice were head-fixed on a stereotaxic frame (SR-5M-HT, Narishige, Tokyo). After partial hair removal and incision of the skin along the midline of the head to expose the skull, two small craniotomies (∼1 mm square for each) were made over the locations of the injection (one for the right CeA, the other for the left PSTh). After the injections, the parietal skin was sutured and glued with a tissue adhesive (Vetbond, 3M, St. Paul, MN). For virus injections into the insula, a mouse was head-fixed on a rotatable head holder (SG-4N, Narishige, Tokyo). After rotating the head 70–80 degrees, the skin of the temporal head (left side) was incised and muscle tissues were peeled to expose the skull. A small craniotomy was performed over the middle-posterior insula at the midpoint between the middle cerebral artery and the vertical squamosal suture immediately dorsal to the ventral eminence of the squamosal bone.^68^ For LED implantations, the craniotomy was performed in the same way as for virus injections into the insula, except for the area of skull removal being a ∼0.8 × 1.2 mm rectangle. Subsequently, a blue tip LED (470 nm, OSBL1608C1A, OptoSupply, Hong Kong) connected with lead wires, the soldered portions of which were insulated with modified silicone resins, was placed over the cranial window so that the LED surface was faced to the exposed cortical surface. The LED was then covered with a silicone adhesive (Kwik-Sil, World Precision Instruments, Sarasota, FL) together with the peeled tissues and skin and further covered with the Kwik-Sil and Vetbond. Using a resin cement (Super-Bond, Sun Medical, Shiga, Japan), the lead wires were fixed on the skull, and a connector was placed on the parietal portion of the skull (see Figure 4A) with a screw embedded into the frontal part of the skull. Immediately after surgery, the mice were injected subcutaneously with buprenorphine (0.1 mg/kg of the body weight, Lepetan, Otsuka Pharmaceutical, Tokyo) and enrofloxacin (5 mg/kg of the body weight, Baytril, Bayer Yakuhin, Tokyo) for postoperative analgesia and blocking of bacterial infections, respectively. All mice were individually housed and maintained in a breeding room on a 12 h light-dark cycle with food and water available *ad libitum*. The mice were housed there for 3 days after CTB injections or 2 weeks after virus injections for axon tracing until sacrifice, and for 4 weeks after virus injections for optogenetics and at least for another 5 days after LED implantations until behavioral experiments.

#### Tracer and virus injections

All injections of tracers and viruses were performed by air pressure (0.05 μl per 1–3 min) with a custom-made syringe pump connected to a glass capillary needle (Drummond precision calibrated micropipettes #2-000-001, ∼40 μm tip diameter) filled with injection solutions. For labeling axonal projections of the insula, an adeno-associated virus (AAV) vector (AAV2-CAG-GFP or AAV2-CAG-FLEX-GFP, University of North Carolina (UNC) Vector Core, 0.2 μl) was injected 0.4–0.5 mm deep from the surface of the middle-posterior insula (0.05 ± 0.3 mm posterior to Bregma, mean ± standard deviation (SD), n = 9 mice: 3 mice for AAV2-CAG-GFP, 6 mice for AAV2-CAG-FLEX-GFP). For bilateral hemispheric labeling of insulae, the AAV2-CAG-GFP and AAV2-CAG-tdTomato (UNC Vector Core, 0.2 μl for each hemisphere) were injected into the left and right insulae, respectively. For retrograde tracing, the CTB conjugated with Alexa Fluor 488 (CTB-green) or 555 (CTB-red) (Invitrogen, approximately 0.05 μl of 5 mg/ml dissolved in 0.1 M phosphate buffer)^69^ was injected into the CeA (1.5 mm posterior, 3.0 mm lateral, 4.5 mm ventral to Bregma, but 0.8 mm posterior, 2.5 mm lateral, 4.9 mm ventral to Bregma for localized injections into the CeM) or the PSTh (2.0 mm posterior, 1.1 mm lateral, 5.0 mm ventral to Bregma). For the mice aged less than 7-week-old, the coordinates of the injection sites were 0.1 mm smaller than the Bregma levels described above. For sublayer-specific protein expressions (i.e., the Cre-inducible GFP, ChR2(H134R)-tdTomato, and tdTomato), retrograde AAVs^36^ described below were injected into either the right CeA or left PSTh. The pAAV-Ef1a-mCherry-IRES-Cre was a gift from Karl Deisseroth (Addgene viral prep # 55632-AAVrg; http://n2t.net/addgene:55632; RRID:Addgene_55632).^66^ The AAV-CAG-hChR2-H134R-tdTomato was a gift from Karel Svoboda (Addgene viral prep # 28017-AAVrg; http://n2t.net/addgene:28017; RRID:Addgene_28017).^46^^,^^67^ The pAAV-CAG-tdTomato (codon diversified) was a gift from Edward Boyden (Addgene viral prep # 59462-AAVrg; http://n2t.net/addgene:59462; RRID:Addgene_59462).

#### Immunohistochemistry

To identify the CeL/CeC, we performed immunohistochemistry against PKC-δ^33^ or the regulator of G protein signaling 14 (RGS14)^70^^,^^71^^,^^72^ as the restricted pattern of RGS14 immunoreactivity to the CeL/CeC was observed in the present study (Figures 3D and 3H). For cortical layer markers, immunohistochemistry against the forkhead box protein P2 (FOXP2) for L6,^73^^,^^74^ NECAB1 for L5a,^34^ and CTIP2 for L5b^35^^,^^36^ was performed. Sections were incubated in PBS containing 5% normal donkey serum and 0.3% Triton X-100 for 1 h at room temperature (RT, 23 ± 7°C) and then stained with primary antibodies overnight at 4°C (rabbit anti-c-Fos, 1:5000, Sigma-Aldrich; rat anti-CTIP2, 1:1000, Abcam; rabbit anti-FOXP2, 1:1000, Abcam; mouse anti-FOXP2, 1:2000, Atlas Antibodies; rat anti-GFP, 1:2000, Nacalai; rabbit anti-NECAB1, 1:2000, Atlas Antibodies; mouse anti-PKC-δ, 1;500, BD Biosciences; mouse anti-RGS14, 1:500, NeuroMab; two overnight incubations for c-Fos and a triple-staining of NECAB1, CTIP2, and FOXP2). After washing three times in PBS for 10 min, the sections were incubated with secondary antibodies for 2 h at RT (donkey anti-mouse IgG Alexa-555 conjugate for PKC-δ and FOXP2; donkey anti-mouse IgG Alexa-647 conjugate for RGS14; donkey anti-rabbit IgG Alexa-488 conjugate for c-Fos; donkey anti-rabbit IgG Alexa-647 conjugate for c-Fos and FOXP2; donkey anti-rabbit IgG Alexa Plus-647 conjugate for NECAB1; donkey anti-rat IgG Alexa-488 conjugate for GFP and CTIP2; donkey anti-rat IgG Alexa Plus-647 conjugate for CTIP2; 1:1000 dilution for all the secondary antibodies, Invitrogen). After washing three times in PBS for 10 min, the sections were mounted on glass slides and counterstained with DAPI. Coverslips were finally mounted with 10% Mowiol 4–88 (Polysciences, Warrington, PA) dissolved in 0.1 M Tris-HCl (pH 8.0) and 25% (w/v) glycerol. The fluorescent images were taken through a confocal laser scanning microscope (FV3000, Olympus, see below) or a fluorescence microscope (BZ-X800, Keyence, Osaka, Japan).

#### Optogenetics

The LED (see surgery) was controlled wirelessly via a receiver unit (TeleR-1-P, TeleOpto, Bio Research Center, Nagoya, Japan) and an infrared emitter (TeleEmitter, Bio Research Center, Nagoya, Japan) connected to a remote controller (TeleRemocon, Bio Research Center, Nagoya, Japan). The controller was operated through a microcontroller (Arduino UNO, Arduino, Somerville, MA) with a custom-made program (on the Arduino IDE). The illumination was a train of 10 pulses (10-ms pulse duration and 50-ms interpulse interval) and was delivered in response to a lick (see Figure 4B). The maximum LED powers (measured before their implantation) were 10 to 20 mW per LED (0.96 mm^2^). For the confirmation of effective optogenetic activations, c-Fos expression in the insula was examined for all mice expressing ChR2 used in the behavioral experiments. To do this, within two weeks of the end of a series of behavioral experiments, the mice were deeply anesthetized and perfused with 4% PFA after repetitive illuminations of the LED for 90 min in a plastic cage (2 s trains of 10 ms pulses at 20 Hz every 4 s), and the brain sections were immunostained with the anti-c-Fos antibody (see immunohistochemistry).

#### Behavioral experiments

To connect a wireless receiver unit immediately before starting a session (including habituation sessions), mice were weakly anesthetized with 4% isoflurane for 2–3 min and then maintained with 2% isoflurane for a minute while the receiver unit was connected to the connector on the head. The mice were then recovered in a home cage for >5 min. Habituation sessions were performed for 3 h on two consecutive days. During the session, the mice (not deprived of water before the sessions) were put in a plastic cage (W 16.5 cm × D 23 cm × H 12 cm, maximum inside dimensions) equipped with a single water spout (Feeding needle KN-348-20G, Natsume Seisakusho, Tokyo) at the height of 4.5 cm from the bottom of the cage, and the cage was placed in a sound-attenuating chamber. Through the spout, a drop of water (approximately 2 μl) was delivered every time the mice licked the spout *ad libitum*. No water was available for 0.5 s after each water drop. The mice were returned to the home cage after the habituation sessions and deprived of water for ∼18 h before starting the test session. A single-spout test session was first carried out for 90 min in which the LED illumination pulse train was delivered in response to a lick in approximately half of the drinking bouts in a pseudorandom order in the session. An interruption of continuous licking for 3 s or longer was considered as the end of a drinking bout. Water was delivered in the same way as in the habituation session regardless of the presence or absence of LED illuminations. Within a week after the single-spout test, two-spout choice tests were performed in which the water-deprived (18 h) mice were put in another plastic cage (W 20 cm × D 31 cm × H 13 cm, maximum inside dimensions) equipped with two water spouts (∼14 cm apart from each other), which delivered equal amount of water (∼2 μl) per lick. A 60 min daily session was performed for two consecutive days. The LED illumination was paired with water delivery from the right spout in the first session, and the pairing was switched to the left spout in the second session to minimize potential spatial bias. Mice with their L5b population expressing ChR2 or tdTomato received a third session (60 min) of two-spout choice test within three days after the second two-spout session. In the third session, the mice were not deprived of water before the test, and the two spouts, one of which (the right side) was paired with the LED illumination, delivered no water upon licking.

For taste-biased two-spout choice control tests, one day after the 3-h habituation in a cage equipped with two water spouts, water-deprived (18 h) B6 intact mice were tested with one spout delivering water and the other delivering equal volume of 0.02% quinine hydrochloride in a 60-min session. Two days later, the same mice (18 h water-deprived) were subjected to a second 60-min session in which one spout delivered water and the other delivered equal volume of 2% sucrose. Seven days after the second session, the mice were further subjected to a third session with both spouts delivering water. For two out of 8 mice, the first test session in which both spouts delivered water was performed one day before the second session with quinine.

Open field (OF) tests were carried out in a 30 cm × 30 cm × 30 cm acrylic chamber placed in a sound-attenuating box. Mice with their L5 population expressing ChR2 or tdTomato were placed in the center of the chamber without water deprivation before the test, and habituated to the chamber for 5 min. LED illumination (1 s trains of 10 ms pulses at 20 Hz every 5 s) was then presented for 2 min, and the illumination was repeated three times every 4 min. The test ended 2 min after the third illumination. The locomotor activity of the mouse was recorded during the test using a video camera (30 frames per sec) above the chamber.

Elevated plus maze (EPM) tests were performed on a custom-made platform composed of two 6 cm × 30 cm open arms, two 6 cm × 30 cm closed arms with 15-cm-high walls, and 6 cm × 6 cm central zone. The platform was elevated 70 cm from the floor of a sound-attenuating room. Mice (non-deprived) whose L5 population expressed ChR2 or tdTomato were placed on the center zone of the platform with no habituation, and the test began within 30 sec thereafter. LED illumination (1 s trains of 10 ms pulses at 20 Hz every 5 s) was presented during the entire session (10 min). The locomotion behavior of the mouse was recorded using a video camera (30 frames per sec) above the maze. The test was aborted if the mouse fell off an open arm: the mice were excluded from analysis if the drop occurred within 8 min from the beginning of the test, otherwise data were collected during the time that elapsed before the drop (i.e. 8–10 min). The EPM tests were carried out on a separate day after spout-licking and OF tests.

### Quantification and statistical analysis

#### Quantitative analyses

To estimate the A-P level of virus injection sites in the insula, serial coronal sections were obtained from each virus-injected brain (n = 3 mice for AAV2.CAG.GFP injections; n = 6 mice for AAV2.CAG.FLEX.GFP injections), and the Bregma level of the section exhibiting the strongest fluorescence was determined by reference to the mouse brain atlas.^75^

To examine the laminar distribution of CTB-labeled cells in the DgI, fluorescent (RGB) images of the coronal sections (the A-P level was between 0.1 mm anterior and 0.2 mm posterior to Bregma) from brains with dual CTB injections (n = 5 mice for CeA-contra/PSTh-ipsi; n = 4 hemispheres from 3 mice for CeA-contra/CeL/CeC-ipsi; n = 4 mice for CeA-contra/CeM-ipsi) were obtained with a confocal laser scanning microscope (10× objective lens, FV3000, Olympus, Tokyo), and z-stack images were created by a maximum intensity projection (ImageJ, NIH) from three confocal slices at 4-μm intervals. Using the ImageJ software, a rectangular region of interest (ROI) (150 μm for the short axis, the long axis was in the direction of cortical depth) was selected in the DgI so that the ROI covered the laminar structure (L1-L6) and the long axis was perpendicular to the border between L5b and L6, indicating cortical depth (layer and subregional boundaries were delineated based on DAPI staining or FOXP2 immunostaining for the L5b-L6 border) (Figures 1A and S1D). The green and red components were then extracted separately as grayscale images from the ROI of the original RGB image. Subsequently, a signal intensity profile along the long axis was obtained by averaging the pixel intensity of the grayscale image across the ROI short axis (150 μm, corresponding to 120 pixels). The fluorescence profile in the L2 to L5b range was extracted, and the values in the profile were scaled to between 0 and 1. Finally, the profile was divided into 20 bins (15–21 pixels per bin except for the last bin with 16–28 pixels), and the bin averages are shown in graphs (Figures 2D, S2B, and S2C). For quantification of CTB-labeled CeA-contra projector and PSTh-ipsi projector cells along the A-P axis of the insula, fluorescent confocal images (z-stack) were obtained from sections at eight different A-P levels (1.1 ± 0.1 mm, 0.8 ± 0.1 mm, 0.5 ± 0.1 mm, 0.2 ± 0.1 mm anterior to Bregma; 0.1 ± 0.1 mm, 0.4 ± 0.1 mm, 0.7 ± 0.1 mm, 1.0 ± 0.1 mm posterior to Bregma), and the number of CTB-labeled cell bodies in the insula was counted for each section (the GI and L6 of the DgI to AgI were excluded from the analysis). The fraction (%) of labeled cells at each A-P level was calculated by the number of labeled cells at each A-P level divided by the sum of labeled cells for all eight levels. Six and five brains were analyzed for CeA-contra projector cells and PSTh-ipsi projector cells, respectively. For the expression of sublayer marker proteins (NECAB1 and CTIP2) in the CTB-labeled cells, fluorescent confocal images (z-stack images created from ten confocal slices at 2-μm intervals) were obtained from coronal sections from brains with dual CTB injections (CeA-contra/PSTh-ipsi, n = 3 mice) stained for either NECAB1 or CTIP2. For each molecular marker, the proportion of the total number of CTB/marker double-positive cells to CTB-labeled cells within the DgI of three proximal sections between 0.2 mm anterior and 0.5 mm posterior to Bregma (L6 was excluded) was calculated.

For the quantification of c-Fos immunoreactive cells after optogenetic activation of the L5 subpopulation, fluorescent confocal images (single slices) were obtained from both opto-stimulated and non-stimulated side of the insula of a coronal section. To isolate significant c-Fos immunoreactive particles using the ImageJ, a binarized image was created from a grayscale image of the c-Fos immunofluorescence as follows: First, a background image was created from the original grayscale image (“Rolling ball radius” of 50 pixels), and the mean gray value and SD of a 400 μm square ROI (the position of which was arbitrarily selected within the region corresponding to the insula) were measured as the background level. The original grayscale image was then binarized by the threshold of the mean + 20 × SD of the background ROI. The binarized image was further filtered with a median filter (radius = 2 pixels), and particles of 20 μm^2^ or larger were regarded as significant c-Fos immunopositive particles. Overlapping particles were identified by reference to the corresponding DAPI image and counted separately. The total number of c-Fos immunopositive particles within the DgI L5 of two proximal sections was counted in each hemisphere as the number of c-Fos-positive cells.

For the quantification of licking behavior, the number of licks was counted for each drinking bout across a session, and the mean and maximum licks per bout (and their ratios between bouts with and without LED illumination), the percentage of bouts with > 10 licks, and the percentage of bouts with LED illumination in the top 10 longest bouts were obtained offline. For two-spout choice tests, in addition to the same analyses as for the single-spout test, counts were also compared between bouts with and without illumination, and also between switched and unswitched choices next to the bouts with illumination.

For OF tests, the locomotor activity of the mouse was traced offline using the ANY-maze software (Stoelting Co., Wood Dale, IL), in which time spent in the center (15 cm square) of the chamber (30 cm square) and the distance traveled were measured for each of three 2 min-illuminated and three 2 min-nonilluminated epochs. The sum of the three epochs (6 min in total) was used to calculate the ratio (illuminated/nonilluminated). For EPM tests, the distance traveled and time spent in the open arms were measured and the data were represented as the percentage of total distance traveled and time of the session, respectively.

#### Statistical analyses

Statistical analyses were performed using R (version 4.0.4, the R Foundation). For comparisons of the fractions of CTB-labeled cells along the A-P axis, the Shapiro-Wilk test was used to test the normality of each data set and the Bartlett’s test was used to determine whether the variances between data sets were equal. Dunnett’s test was used to determine whether the peak (maximum) of the fractions is significantly different from other values. The means of the maximum fractions (0.4 ± 0.1 mm posterior to Bregma for the L5a population; 1.1 ± 0.1 mm anterior to Bregma for the L5b population) were used as the control group in Dunnett’s test. For optogenetic and behavioral experiments, the Shapiro-Wilk test was used to test the normality of each dataset (the difference between pairs was tested for pairwise comparisons). F-test was further used to test if the variances of two unpaired groups were equal. For comparisons between two unpaired groups, Student’s *t* test was used for groups with both normal distributions and homogeneity of variances, Welch’s t-test was used for groups with normal distributions but no significant equality of variances, and Wilcoxon rank sum (Mann–Whitney U) test was used for pairs involving one or two non-normally distributed groups. For comparisons between paired groups, paired t-test was used if the difference values between groups were normally distributed; otherwise, Wilcoxon signed rank test was used. For multiple comparisons, one-way ANOVA and Welch’s ANOVA were used if the variances of groups with normal distributions were homogeneous and heterogeneous, respectively. The Steel-Dwass' tests were used for groups involving one or more non-normally distributed data. p < 0.05 was considered statistically significant. Data are expressed as mean ± standard error of the mean unless otherwise stated.

Detailed information on the materials used in this study is listed in Table S1.

## Data Availability

• All data reported in this paper are listed in Data S1. • This paper does not report original code. • Any additional information required to reanalyze the data reported in this paper is available from the lead contact upon request.

## Supplemental information

Document S1. Figures S1–S7 and Table S1

Data S1. List of datasets and the statistical analyses in main and supplementary figures
