# Supplementary material for: Dissection of insular cortex layer 5 reveals two sublayers with opposing modulatory roles in appetitive drinking behavior
Source: iScience. 2023 May 26;26(6):106985. doi: 10.1016/j.isci.2023.106985 (PMC10291511; doi:10.1016/j.isci.2023.106985)
Supplement: Document S1. Figures S1–S7 and Table S1 [file mmc1.pdf]

iScience, Volume *26*

Supplemental information

Dissection of insular cortex layer 5 reveals two sublayers with opposing modulatory roles in appetitive drinking behavior

Makoto Takemoto, Shigeki Kato, Kazuto Kobayashi, and Wen-Jie Song

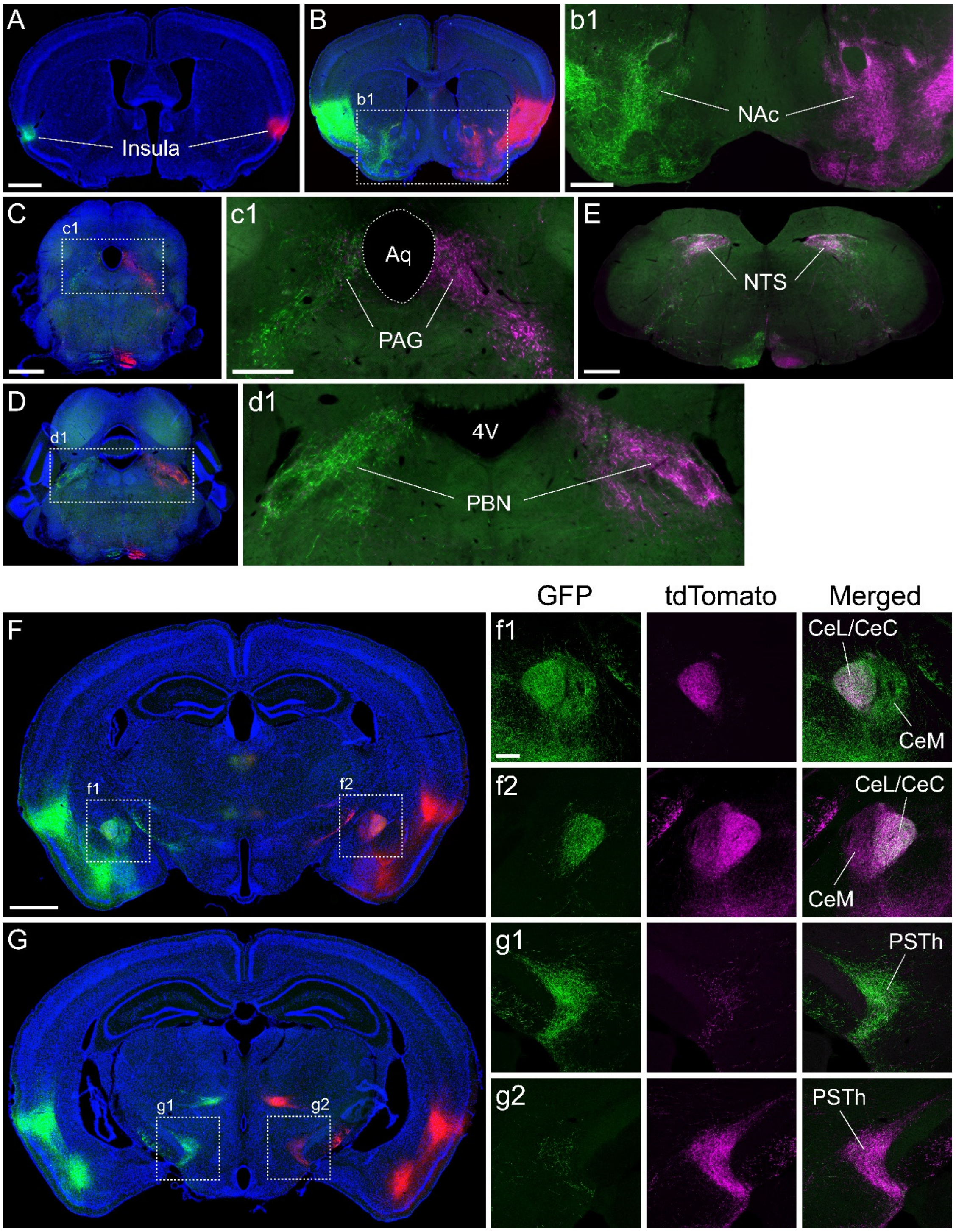

Figure S1, related to Figure 1. Subcortical projections of the insula. (A) A typical example image of the coronal section containing virus injection sites in the left (AAV2-GFP, green) and right (AAV2-tdTomato, red) insulae. (B) An image of the section containing the nucleus accumbens (NAc) with an enhanced fluorescence intensity. (b1) A magnified view of the box in B.

(C) An image of the section containing the periaqueductal gray (PAG). (c1) A magnified view of the box in C. Aq: aqueduct. (D) An image of the section containing the parabrachial nucleus (PBN). (d1) A magnified view of the box in D. 4V: 4th ventricle. (E) An image of the section containing the nucleus of the solitary tract (NTS). (F) An image of the section containing the central amygdala. (f1, f2) Higher magnification views of the boxes in the left (f1) and right (f2) hemispheres of the section in F. Left, GFP; middle, tdTomato; right, merged. Note that the projections of GFP-expressing axons and tdTomato-expressing axons overlapped in the CeL/CeC but not in the CeM. (G) An image of the section containing the parasubthalamic nucleus (PSTh). (g1, g2) Higher magnification views of the boxes in the left (g1) and right (g2) hemispheres of the section in G. Left, GFP; middle, tdTomato; right, merged. Note few of the tdTomato-expressing axons (g1-middle) and GFP-expressing axons (g2-left) project contralaterally to the PSTh. The images in A-D, F, G were merged with DAPI (blue). Scale bars: 1 mm (A, C, F), 0.5 mm (b1, c1, E), 0.2 mm (f1).

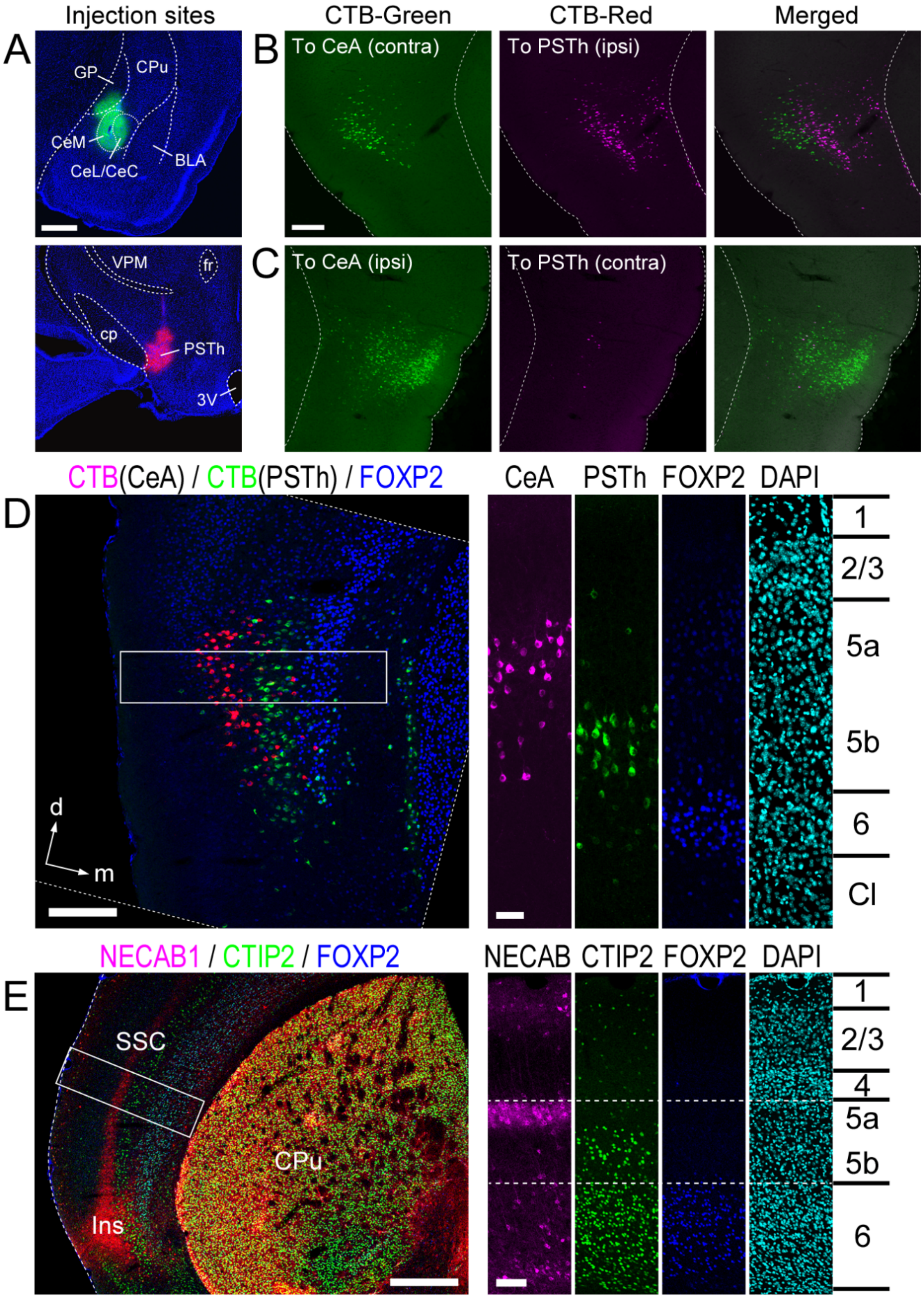

Injection sites
CTB-Green
CTB-Red
Merged
A
GP
CPu
CeM
CeL/CeC
BLA
VPM
fr
cp
PSTh
3V
B
To CeA (contra)
To PSTh (ipsi)
C
To CeA (ipsi)
To PSTh (contra)
CTB(CeA) / CTB(PSTh) / FOXP2
D
d
m
CeA
PSTh
FOXP2
DAPI
1
2/3
5a
5b
6
Cl
NECAB1 / CTIP2 / FOXP2
E
SSC
CPu
Ins
NECAB
CTIP2
FOXP2
DAPI
1
2/3
4
5a
5b
6

Figure S2, related to Figure 2. Distribution of insula cells retrogradely labeled by dual CTB injections into the right CeA and the left PSTh. (A) Typical example images of the injection sites in the right CeA (top, CTB-green) and the left PSTh (bottom, CTB-red). Sections were counterstained with DAPI (blue). BLA, basolateral amygdala; cp, cerebral peduncle; CPu, caudate-putamen; fr, fasciculus retroflexus; GP, globus pallidus; VPM, ventroposterior medial nucleus of the thalamus, the medial part; 3V, third ventricle. (B) Typical example images of the left insula containing CeA-contra-projecting (green) and PSTh-ipsi-projecting (magenta) CTB-labeled cells. The left and middle images are the same as shown in Fig. 2C (left and middle), and the right image is a merged one. (C) Typical example images of the right insula from the same brain as in B containing CeA-ipsi-projecting (green) and PSTh-contra-projecting (magenta) CTB-labeled cells. Note that CeA-ipsi-projecting labeled cells (C, green) were distributed more broadly than CeA-contra-projecting labeled cells (B, green) and that only a few PSTh-contra-projecting labeled cells (C, magenta) were seen. (D) Sublayer localization of CeA-contra-projecting (red/magenta) and PSTh-ipsi-projecting (green) CTB-labeled cells in L5 of the DgI. The left image was rotated clockwise at 14 degrees (dotted lines). The right images are higher magnification views of the box in the left merged image shown in separated fluorescent colors and DAPI staining (cyan). The border between L5 and L6 can be delineated based on the difference of DAPI-stained cell density and on immunostaining for an L6 marker, FOXP2 (blue). Cl, claustrum; d, dorsal; m, medial. (E) Layer-specific expressions of NECAB1 (red/magenta), CTIP2 (green), and FOXP2 (blue) in the somatosensory cortex (SSC) and insula (Ins). The right images are higher magnification views of the boxed region (a part of the SSC) in the left merged image shown in separated fluorescent colors and DAPI staining (cyan). Note that NECAB1+ cells and CTIP2+ cells are clearly segregated within L5 although both molecular expressions are also found in superficial layers and L6. Scale bars: 0.5 mm (A and E-left), 0.2 mm (B and D-left), 0.1 mm (E-right), 50 μm (D-right).

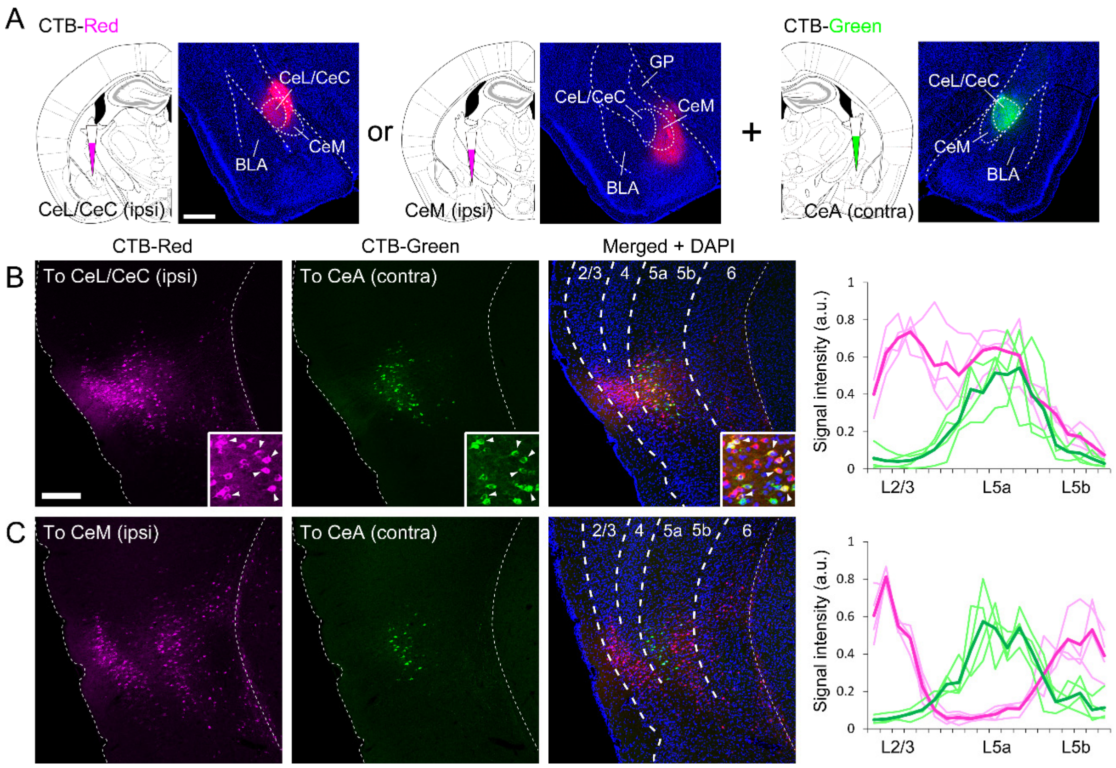

Figure S3, related to Figure 2A-D. Layer-specific DgI projections to the CeA. (A) Illustrations depicting the injection of CTB-red preferentially into the CeL/CeC or CeM of the left hemisphere, accompanied by the injection of CTB-green into the right CeA. The fluorescent images are typical examples showing the CTB injection sites (counterstained with DAPI) corresponding to the adjacent illustrations. (B) Typical example images of the left insula containing CeL/CeC-ipsi-projecting (red/magenta) and CeA-contra-projecting (green) CTB-labeled cells. The right image shows the merged fluorescence (counterstained with DAPI). Double-labeled cells can be seen in L5a (arrowheads in the insets). Right: relative fluorescent intensities (arbitrary unit) of CTB labeling across cortical depth in the DgI (L2/3 to L5b). The signal distributions of CTB-red-labeled cells (projecting to the CeL/CeC-ipsi) and CTB-green-labeled cells (projecting to the CeA-contra) are shown in magenta and green, respectively. Light colors represent individual mice (n = 4 mice with dual CTB injections), and dark colors are the average. Note that peaks are found in L2/3 and L5a for CeL/CeC-ipsi-projecting cells (magenta). (C) Typical example images of the left insula containing CeM-ipsi-projecting (red/magenta) and CeA-contra-projecting (green) CTB-labeled cells. The right image shows the merged fluorescence (counterstained with DAPI). Right: relative fluorescent intensities (arbitrary unit) of CTB labeling across cortical depth in the DgI (L2/3 to L5b). The signal distributions of CTB-red-labeled cells (projecting to the CeM-ipsi) and CTB-green-labeled cells (projecting to the CeA-contra) are shown in magenta and green, respectively. Light colors represent individual mice (n = 4 mice with dual CTB injections), and dark colors are the average. Note that peaks are found in L2/3 and L5b but not in L5a for CeM-ipsi-projecting cells (magenta). Scale bars: 0.5 mm (A), 0.2 mm (B).

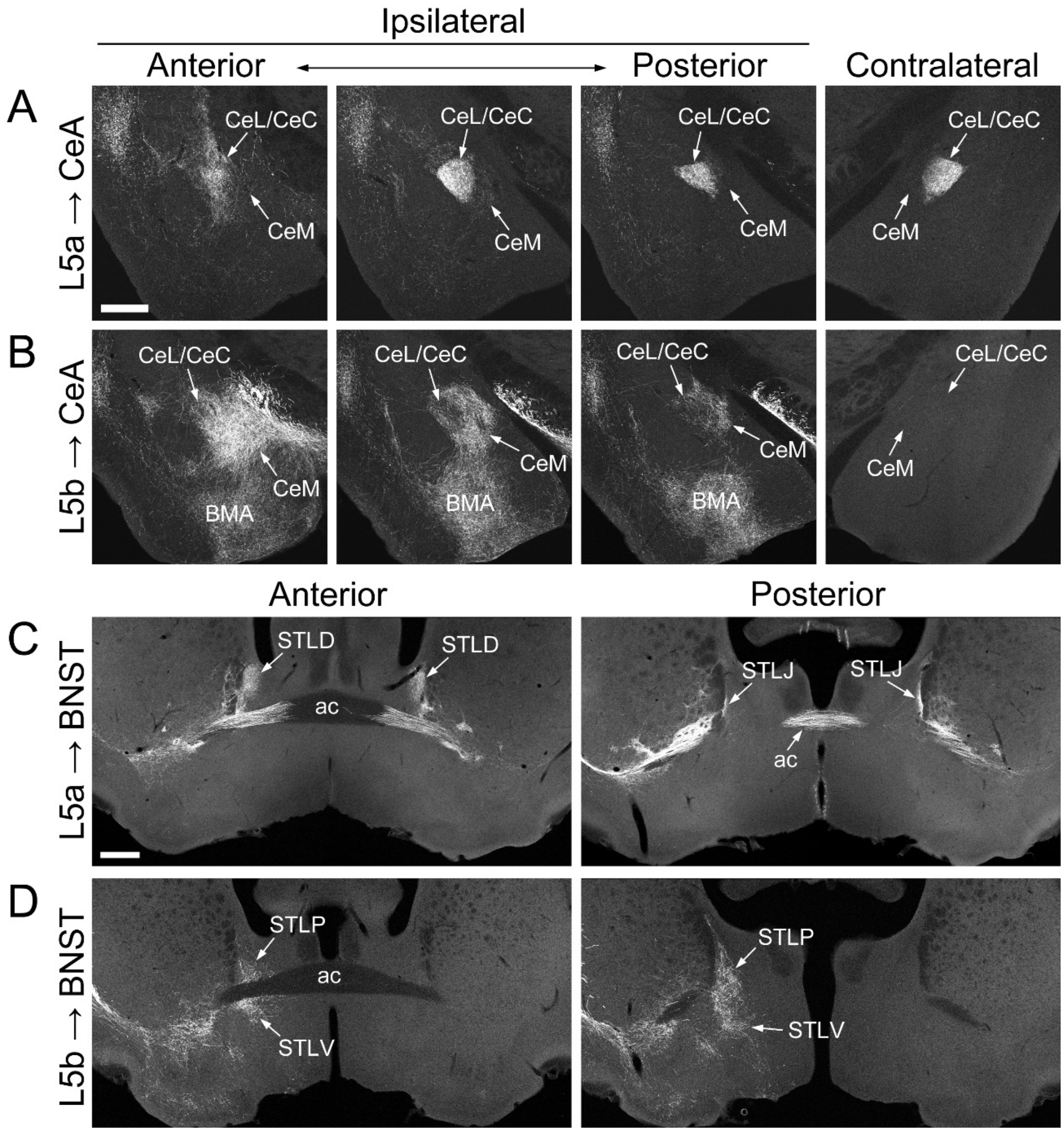

Figure S4, related to Figure 3. Differential organizations of axonal projections from sublaminar neuronal populations in L5 of the insula to the extended amygdala. (A, B) GFP-expressing axons of the L5a (A) and L5b (B) neuronal populations projecting to the CeA. The ipsilateral CeA at three levels along the A-P axis and a middle part of the contralateral CeA are shown. Note the absence of L5b-derived labeled axons in the contralateral amygdala (B). BMA, basomedial amygdala. (C, D) GFP-expressing axons of the L5a (C) and L5b (D) neuronal populations projecting to the BNST. Two sections along the A-P axis are shown. Note the absence of L5b-derived labeled axons running through the anterior commissure (ac) and projecting to the contralateral BNST (D). Gray-scale images are shown. Scale bars: 0.5 mm.

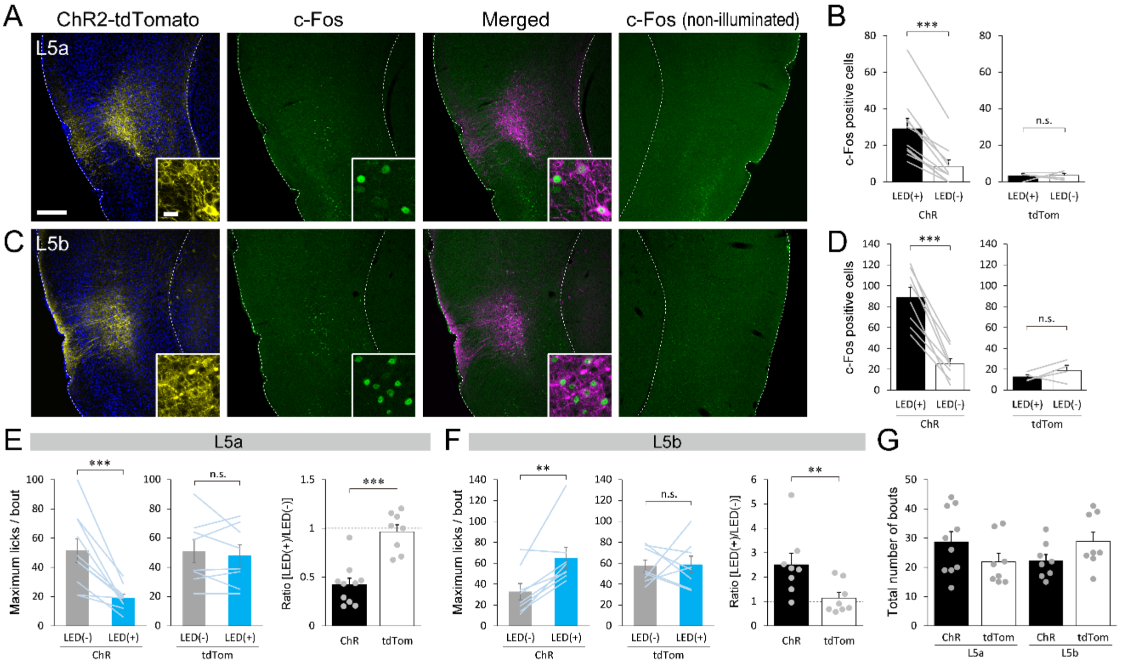

Figure S5, related to Figure 4. Sublayer-specific optogenetic activation of the insula and its effect on appetitive licking. (A, C) c-Fos expression in ChR2-expressing L5 cell populations induced by LED illumination of the insula. The expression of ChR2-tdTomato fusion protein (yellow and magenta) and c-Fos (green) in L5a (A) and L5b (C) neuronal subpopulations of the left insula. Few c-Fos immunoreactive cell nuclei are seen in the right (non-illuminated) insula. Scale bars: 0.2 mm, 20 μm (inset). (B, D) Quantification of the number of c-Fos immunoreactive cells (sum of the number of cells from two sections per hemisphere for each mouse) in the illuminated [LED(+)] vs. non-illuminated [LED(-)] side of the insula for ChR2-expressing mice (L5a: 29.0 ± 5.7 for [LED(+)] vs. 8.5 ± 3.4 for [LED(-)], Paired t-test, $p < 0.001$, n = 10 mice, B-left; L5b: 89.1 ± 9.3 for [LED(+)] vs. 25.0 ± 5.2 for [LED(-)], Paired t-test, $p < 0.001$, n = 8 mice, D-left) and tdTomato-expressing control mice (L5a: 3.3 ± 1.1 for [LED(+)] vs. 3.5 ± 1.2 for [LED(-)], Paired t-test, $p = 0.91$, n = 4 mice, B-right; L5b: 12.8 ± 2.1 for [LED(+)] vs. 19.0 ± 4.9 for [LED(-)], Paired t-test, $p = 0.29$, n = 4 mice, D-right). Gray lines represent individual mice. (E, F) The maximum number of licks per bout in the single-spout test. Left: comparisons between the bouts with (blue) and without (gray) illumination for mice expressing ChR2 or tdTomato in the L5a population (ChR: 51.5 ± 8.0 for [LED(-)] vs. 19.0 ± 2.5 for [LED(+)], Paired t-test, $p < 0.001$, E-left; tdTom: 51.1 ± 8.0 for [LED(-)] vs. 48.3 ± 7.3 for [LED(+)], Paired t-test, $p = 0.54$, E-middle) and L5b population (ChR: 32.8 ± 7.8 for [LED(-)] vs. 64.8 ± 10.3 for [LED(+)], Paired t-test, $p = 0.0055$, F-left; tdTom: 57.3 ± 5.9 for [LED(-)] vs. 58.8 ± 7.9 for [LED(+)], Paired t-test, $p = 0.90$, F-middle). Right: the ratio of the maximum number of licks per bout with illumination relative to those without illumination (L5a: 0.42 ± 0.07 for ChR vs. 0.97 ± 0.07 for tdTom, Student's t-test, $p < 0.001$, E; L5b: 2.51 ± 0.47 for ChR vs. 1.14 ± 0.23 for tdTom, Wilcoxon rank sum test, $p < 0.01$, F). Lines and gray dots represent individual mice (L5a: n = 10 for ChR, n = 8 for tdTom, E; L5b: n = 8 for ChR, n = 8 for tdTom, F). Dotted lines indicate the chance level. (G) Total number of drinking bouts in the single-spout session (L5a: 28.7 ± 3.5 for ChR, 21.9 ± 2.9 for tdTom; L5b: 22.3 ± 2.1 for ChR, 28.9 ± 3.2 for tdTom; Steel-Dwass' test: $p = 0.60$ for L5a-ChR vs. L5a-tdTom, $p = 0.63$ for L5a-ChR vs. L5b-ChR, $p = 1.00$ for L5a-ChR vs. L5b-tdTom, $p = 0.96$ for L5a-tdTom vs. L5b-ChR, $p = 0.21$ for L5a-tdTom vs. L5b-tdTom, $p = 0.36$ for L5b-ChR vs. L5b-tdTom). Dots represent individual mice (L5a: n = 10 for ChR, n = 8 for tdTom; L5b: n = 8 for ChR, n = 8 for tdTom). ** $p < 0.01$, *** $p < 0.001$.

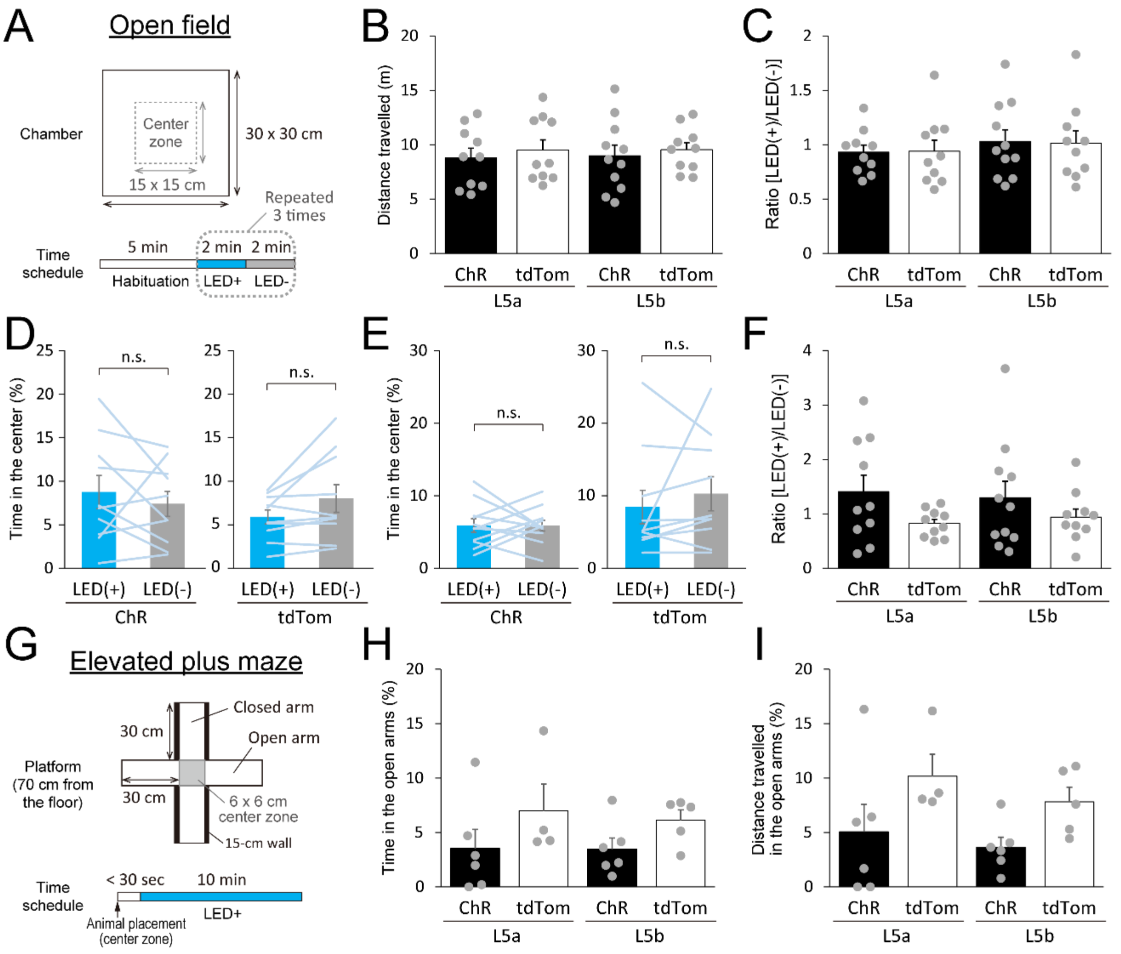

Figure S6. The effect of optogenetic activation of L5 subpopulations on general locomotor activities and anxiety-related behavior. (A) Experimental design of the open field test. (B) The total distance traveled during LED illumination (8.8 ± 0.9 m for L5a-ChR, 9.5 ± 0.9 m for L5a-tdTom, 9.0 ± 1.0 m for L5b-ChR, 9.5 ± 0.6 m for L5b-tdTom, one-way ANOVA, $F(3,37) = 0.18$, $p = 0.91$). (C) The ratio of the total distance traveled during the periods with illumination (LED+) relative to that during the periods without illumination (LED-) (0.93 ± 0.06 for L5a-ChR, 0.94 ± 0.10 for L5a-tdTom, 1.03 ± 0.11 for L5b-ChR, 1.02 ± 0.11 for L5b-tdTom, one-way ANOVA, $F(3,37) = 0.27$, $p = 0.85$). (D) The percentage of time spent in the center of the chamber during illumination vs. during no illumination for L5a (ChR: 8.8 ± 1.9% for LED+ vs. 7.4 ± 1.4% for LED-, Paired t-test, $p = 0.44$, left; tdTom: 5.9 ± 0.8% for LED+ vs. 8.0 ± 1.6% for LED-, Wilcoxon signed rank test, $p = 0.084$, right). (E) The percentage of time spent in the center of the chamber during illumination vs. during no illumination for L5b (ChR: 5.9 ± 0.9% for LED+ vs. 5.9 ± 0.8% for LED-, Paired t-test, $p = 0.97$, left; tdTom: 8.5 ± 2.3% for LED+ vs. 10.3 ± 2.4% for LED-, Wilcoxon signed rank test, $p = 0.38$, right). (F) The ratio of time spent in the center of the chamber between LED+ and LED- (1.41 ± 0.30 for L5a-ChR, 0.82 ± 0.08 for L5a-tdTom, 1.30 ± 0.30 for L5b-ChR, 0.94 ± 0.15 for L5b-tdTom, Welch's ANOVA, $F(3,18.4) = 1.75$, $p = 0.19$). Lines in D and E and dots in B, C, and F represent individual mice (L5a: n = 10 for ChR, n = 10 for tdTom; L5b: n = 11 for ChR, n = 10 for tdTom). (G) Experimental design of the elevated plus maze test. (H) The percentage of time spent in the open arms across the session (3.5 ± 1.7% for L5a-ChR, 7.0 ± 2.5% for L5a-tdTom, 3.5 ± 1.0% for L5b-ChR, 6.1 ± 0.9% for L5b-tdTom; Steel-Dwass' test: $p = 0.44$ for L5a-ChR vs. L5a-tdTom, $p = 0.96$ for L5a-ChR vs. L5b-ChR, $p = 0.46$ for L5a-ChR vs. L5b-tdTom, $p = 0.26$ for L5a-tdTom vs. L5b-ChR, $p = 0.99$ for L5a-tdTom vs. L5b-tdTom, $p = 0.46$ for L5b-ChR vs. L5b-tdTom). (I) The percentage of distance traveled in the open arms across the

session (5.1 ± 2.5% for L5a-ChR, 10.2 ± 2.0% for L5a-tdTom, 3.6 ± 0.9% for L5b-ChR, 7.8 ± 1.4% for L5b-tdTom; Steel-Dwass' test: $p = 0.32$ for L5a-ChR vs. L5a-tdTom, $p = 1.00$ for L5a-ChR vs. L5b-ChR, $p = 0.69$ for L5a-ChR vs. L5b-tdTom, $p = 0.051$ for L5a-tdTom vs. L5b-ChR, $p = 0.76$ for L5a-tdTom vs. L5b-tdTom, $p = 0.13$ for L5b-ChR vs. L5b-tdTom). Dots represent individual mice (L5a: $n = 6$ for ChR, $n = 4$ for tdTom; L5b: $n = 6$ for ChR, $n = 5$ for tdTom).

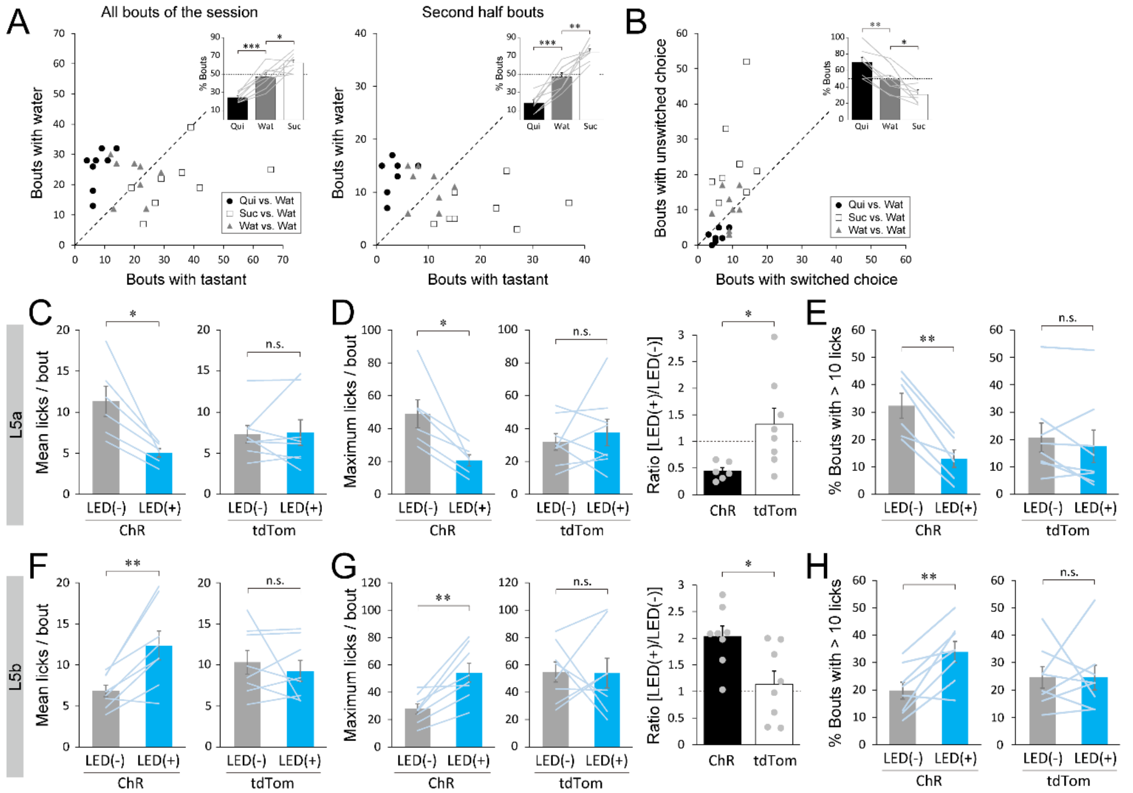

Figure S7, related to Figure 5. The effect of optogenetic activation of L5 subpopulations on appetitive licking in two-spout choice test. (A) Left: the number of bouts in the whole session in which the B6 intact mouse chose the spout with water vs. tastants (quinine [Qui, black circles], sucrose [Suc, white squares], water [Wat, gray triangles]) (Qui vs Wat: 7.9 ± 1.2 for quinine vs. 25.6 ± 2.4 for water, Paired t-test, $p < 0.001$; Suc vs Wat: 35.1 ± 5.2 for sucrose vs. 21.1 ± 3.3 for water, Paired t-test, $p = 0.021$; Wat vs Wat: 19.5 ± 2.1 for spout-A vs. 22.3 ± 2.5 for spout-B, Paired t-test, $p = 0.45$). The inset bar graph represents the percentage of bouts with tastant spouts (24.3 ± 1.8% for Qui, 47.1 ± 4.3% for Wat, 62.4 ± 3.5% for Suc, Paired t-test, $p < 0.001$ for Qui vs. Wat, Paired t-test, $p = 0.029$ for Suc vs. Wat). Right: the same quantification within the second half bouts of the session (Qui vs Wat: 3.1 ± 0.8 for quinine vs. 13.4 ± 1.2 for water, Paired t-test, $p < 0.001$; Suc vs Wat: 20.9 ± 3.1 for sucrose vs. 7.0 ± 1.3 for water, Paired t-test, $p < 0.01$; Wat vs Wat: 9.6 ± 1.2 for spout-A vs. 11.0 ± 1.3 for spout-B, Paired t-test, $p = 0.51$). The inset bar graph represents the percentage of bouts with tastant spouts (18.2 ± 3.3% for Qui, 47.0 ± 4.7% for Wat, 74.4 ± 3.3% for Suc, Paired t-test, $p < 0.001$ for Qui vs. Wat, Paired t-test, $p < 0.01$ for Suc vs. Wat). N = 8 mice. (B) The number of bouts in which the mouse (B6 intact) switched vs. unswitched choice next to the bouts with tastants (Qui vs Wat: 5.3 ± 0.7 for switched spout vs. 2.6 ± 0.6 for unswitched spout, Paired t-test, $p < 0.01$; Suc vs Wat: 10.3 ± 1.6 for switched spout vs. 24.1 ± 4.6 for unswitched spout, Paired t-test, $p = 0.015$; Wat vs Wat: 9.0 ± 0.9 for switched spout vs. 10.4 ± 1.9 for unswitched spout, Paired t-test, $p = 0.51$). The inset bar graph represents the percentage of bouts with tastant spouts (69.8 ± 6.2% for Qui, 48.9 ± 5.9% for Wat, 30.8 ± 4.0% for Suc, Paired t-test, $p < 0.01$ for Qui vs. Wat, Paired t-test, $p = 0.020$ for Suc vs. Wat). N = 8 mice. (C, F) Comparisons of mean licks per bout between the bouts with (blue) and without (gray) illumination for mice expressing ChR2 or tdTomato in the L5a population (ChR: 11.3 ± 1.8 for [LED-] vs. 5.0 ± 0.5 for [LED+], Paired t-test, $p = 0.010$, C-left; tdTom: 7.3 ± 1.1 for [LED-] vs. 7.5 ± 1.6 for [LED+], Paired t-test, $p = 0.87$, C-right) and L5b population (ChR: 6.8 ± 0.7 for [LED-] vs. 12.4 ± 1.8 for [LED+], Paired t-test, $p < 0.01$, F-left; tdTom: 10.3 ± 1.4 for [LED-] vs. 9.3 ± 1.3 for [LED+], Wilcoxon signed rank test, $p = 0.95$, F-right). (D, G) The maximum number of licks per bout in the two-spout choice test. Left: comparisons between the bouts with (blue) and without

(gray) illumination for mice expressing ChR2 or tdTomato in the L5a population (ChR: 49.0 ± 8.6 for [LED-] vs. 20.7 ± 3.5 for [LED+], Wilcoxon signed rank test, $p = 0.031$, D-left; tdTom: 31.9 ± 5.1 for [LED-] vs. 37.6 ± 8.1 for [LED+], Paired t-test, $p = 0.51$, D-middle) and L5b population (ChR: 27.9 ± 3.7 for [LED-] vs. 54.3 ± 6.9 for [LED+], Paired t-test, $p < 0.01$, G-left; tdTom: 54.8 ± 7.4 for [LED-] vs. 53.9 ± 10.8 for [LED+], Paired t-test, $p = 0.95$, G-middle). Right: the ratio of the maximum number of licks per bout with illumination relative to those without illumination (L5a: 0.44 ± 0.07 for ChR vs. 1.33 ± 0.30 for tdTom, Welch's t-test, $p = 0.021$, D; L5b: 2.04 ± 0.19 for ChR vs. 1.14 ± 0.25 for tdTom, Student's t-test, $p = 0.013$, G). (E, H) Proportion of long drinking bouts with > 10 licks per bout in the sessions (L5a-ChR: 32.3 ± 4.6% for [LED-] vs. 13.0 ± 3.2% for [LED+], Paired t-test, $p < 0.01$, E-left; L5a-tdTom: 20.7 ± 5.3% for [LED-] vs. 17.5 ± 5.9% for [LED+], Paired t-test, $p = 0.26$, E-right; L5b-ChR: 19.8 ± 3.1% for [LED-] vs. 33.9 ± 3.8% for [LED+], Paired t-test, $p < 0.01$, H-left; L5b-tdTom: 24.6 ± 4.0% for [LED-] vs. 24.5 ± 4.5% for [LED+], Paired t-test, $p = 1.00$, H-right). Lines and gray dots represent individual mice (L5a: $n = 6$ for ChR, $n = 8$ for tdTom in C-E; L5b: $n = 8$ for ChR, $n = 8$ for tdTom in F-H). Dotted lines in A, B, D (right), and G (right) indicate the chance level. * $p < 0.05$, ** $p < 0.01$, *** $p < 0.001$.

Reagent, tool, and resource	Source	Identifier	Concentration/dilution/titer
Drugs and reagents			
Ketamine injection 5%	Fujita Pharmaceutical Co., Ltd., Tokyo	N/A	
Selactar 2% Injection	Bayer Yakuhin, Tokyo	N/A	
Lepetan injection 0.2^{mg}	Otsuka Pharmaceutical Co., Ltd., Tokyo	N/A	
Baytril 5% Injection	Bayer Yakuhin, Tokyo	N/A	
Cholera Toxin Subunit B (Recombinant), Alexa Fluor™ 488 Conjugate	Thermo Fisher Scientific	C22841	0.5% (in 0.1 M phosphate buffer)
Cholera Toxin Subunit B (Recombinant), Alexa Fluor™ 555 Conjugate	Thermo Fisher Scientific	C22843	0.5% (in 0.1 M phosphate buffer)
Mowiol® 4-88	Polysciences, Inc.	17951	
Antibodies			
Anti-c-Fos, rabbit polyclonal	Sigma-Aldrich	F7799	1:5000
Anti-CTIP2, rat monoclonal [25B6]	Abcam	ab18465	1:1000
Anti-FOXP2, rabbit polyclonal	Abcam	ab16046	1:1000
Anti-FOXP2, mouse monoclonal	Atlas Antibodies	AMAb91361	1:2000
Anti-GFP, rat monoclonal [GF090R]	Nacalai Tesque Inc., Kyoto Japan	04404-84	1:2000
Anti-NECAB1, rabbit polyclonal	Atlas Antibodies	HPA023629	1:2000
Anti-PKCδ, mouse monoclonal	BD Biosciences	610398	1:500
Anti-RGS14, mouse monoclonal [NeuroMab clone N133/21]	NeuroMab, Davis CA	75-170	1:500
Donkey anti-Rat IgG (H+L) Highly Cross-Adsorbed Secondary Antibody, Alexa Fluor 488	Thermo Fisher Scientific	A-21208	1:1000
Donkey Anti-Mouse IgG H&L (Alexa Fluor® 647) preadsorbed	Abcam	ab150111	1:1000
Donkey Anti-Rabbit IgG H&L (Alexa Fluor® 647) preadsorbed	Abcam	ab150063	1:1000 (for FOXP2)
Donkey anti-Rabbit IgG (H+L) Highly Cross-Adsorbed Secondary Antibody, Alexa Fluor Plus 647	Thermo Fisher Scientific	A32795	1:1000 (for c-Fos)
Donkey anti-Rabbit IgG (H+L) Highly Cross-Adsorbed Secondary Antibody, Alexa Fluor 488	Thermo Fisher Scientific	A-21206	1:1000 (for c-Fos)
Viruses			
rAAV2-CAG-GFP	UNC Vector Core (Edward Boyden)	N/A	4.3x10^12 (qPCR titer vg/ml)
rAAV2-CAG-FLEX-GFP	UNC Vector Core (Edward Boyden)	N/A	3.7x10^12 (qPCR titer vg/ml)
pAAV-Ef1a-mCherry-IRES-Cre (AAV Retrograde)	Addgene (Karl Deisseroth)	55632-AAVrg	1.3x10^13 (GC/ml)
AAV-CAG-hChR2-H134R-tdTomato (AAV Retrograde)	Addgene (Karel Svoboda)	28017-AAVrg	9.0x10^12 - 1.0x10^13 (GC/ml)
pAAV-CAG-tdTomato (codon diversified) (AAV Retrograde)	Addgene (Edward Boyden)	59462-AAVrg	1.0x10^13 (GC/ml)
Mouse			
C57BL/6JJmsSlc	Japan SLC, Shizuoka Japan	N/A	
Softwares			
ImageJ 1.52a	NIH	https://imagej.nih.gov/ij/	
Arduino IDE 1.8.12	Arduino.cc	https://www.arduino.cc/en/software	
R version 4.0.4	The R Foundation	https://www.r-project.org/	
ANY-maze v6.17 and v7.16	Stoelting Co.	https://www.any-maze.com/	
Tools for optogenetics and behavior			
Chip LED (470 nm)	OptoSupply Ltd, N.T., Hong Kong	OSBL1608C1A	
Teleopto receiver	Bio Research Center Co., Ltd., Aichi Japan	TeleR-1-P	
Teleopto infrared emitter	Bio Research Center Co., Ltd., Aichi Japan	TeleEmitter	
Teleopto controller	Bio Research Center Co., Ltd., Aichi Japan	TeleRemocon	
Arduino UNO Rev3	Arduino, Somerville, MA	A000066	
Feeding needle	Natsume Seisakusho Co., Ltd., Tokyo Japan	KN-348-20G-50	
Kwik-Sil™ silicone adhesive	World Precision Instruments, Inc., Sarasota FL	KWIK-SIL	
Vetbond	3M, St. Paul, MN	1469SB	
Super-Bond	Sun Medical Co., Ltd., Shiga, Japan	Super-Bond C&B	

Table S1. List of reagents, tools, and resources used in this study, related to the STAR Methods.
